# CCL5 promotion of bioenergy metabolism is crucial for hippocampal synapse complex and memory formation

**DOI:** 10.1038/s41380-021-01103-3

**Published:** 2021-04-30

**Authors:** Reni Ajoy, Yu-Chun Lo, Man-Hau Ho, You-Yin Chen, Yun Wang, Yuan-Hao Chen, Chiu Jing-Yuan, Chun Austin Changou, Yuan-Chin Hsiung, Hui-Min Chen, Tzu-Hao Chang, Cheng-Yang Lee, Yung-Hsiao Chiang, Wen-Chang Chang, Barry Hoffer, Szu-Yi Chou

**Affiliations:** 1 Ph.D. Program for Neural Regenerative Medicine, College of Medical Science and Technology, Taipei Medical University and National Health Research Institutes, Taipei, Taiwan; 2 Graduate Institute of Neural Regenerative Medicine, College of Medical Science and Technology, Taipei Medical University, Taipei, Taiwan; 3 Graduate Institute of Medical Sciences, College of Medicine, Taipei Medical University, Taipei, Taiwan; 4 Department of Biomedical Engineering, National Yang Ming University, No.155, Sec.2, Taipei, Taiwan; 5 Department of Biomedical Engineering, National Yang Ming Chiao Tung University, Hsinchu, Taiwan; 6 Center for Neuropsychiatric Research, National Health Research Institutes, Zhunan, Miaoli County, Taiwan; 7 Department of Neurological Surgery, Tri-Service General Hospital, National Defense Medical Center, Taipei, Taiwan; 8 The Ph.D. Program for Translational Medicine, College of Medical Science and Technology, Taipei Medical University and Academia Sinica, Taipei Medical University and Academia Sinica, Taipei, Taiwan; 9 The Ph.D. Program for Cancer Molecular Biology and Drug Discovery, College of Medical Science and Technology, Taipei Medical University and Academia Sinica, Taipei Medical University, Taipei, Taiwan; 10 Core Facility, Taipei Medical University, Taipei, Taiwan; 11 Department of Anatomy and Cell Biology, School of Medicine, College of Medicine, Taipei Medical University, Taipei, Taiwan; 12 Graduate Institute of Biomedical Informatics, Taipei Medical University, Taipei, Taiwan; 13 Research Information Session, Office of Information Technology, Taipei Medical University, Taipei, Taiwan; 14 Department of Surgery, School of Medicine, College of Medicine, Taipei Medical University, Taipei, Taiwan; 15 Department of Neurosurgery, Taipei Medical University Hospital, Taipei, Taiwan; 16 Neuroscience Research Center, Taipei Medical University, Taipei, Taiwan; 17 Department of Neurosurgery, Case Western Reserve University School of Medicine, Cleveland, OH USA; 18 Scientist Emeritus, National Institutes of Health, Bethesda, MD USA

**Keywords:** Neuroscience, Physiology

## Abstract

Glucoregulatory efficiency and ATP production are key regulators for neuronal plasticity and memory formation. Besides its chemotactic and neuroinflammatory functions, the CC chemokine––CCL5 displays neurotrophic activity. We found impaired learning-memory and cognition in CCL5-knockout mice at 4 months of age correlated with reduced hippocampal long-term potentiation and impaired synapse structure. Re-expressing CCL5 in knockout mouse hippocampus restored synaptic protein expression, neuronal connectivity and cognitive function. Using metabolomics coupled with FDG-PET imaging and seahorse analysis, we found that CCL5 participates in hippocampal fructose and mannose degradation, glycolysis, gluconeogenesis as well as glutamate and purine metabolism. CCL5 additionally supports mitochondrial structural integrity, purine synthesis, ATP generation, and subsequent aerobic glucose metabolism. Overexpressing CCL5 in WT mice also enhanced memory-cognition performance as well as hippocampal neuronal activity and connectivity through promotion of de novo purine and glutamate metabolism. Thus, CCL5 actions on glucose aerobic metabolism are critical for mitochondrial function which contribute to hippocampal spine and synapse formation, improving learning and memory.

## Abstract (web summary)

In addition to neuroinflammatory functions, chemokine CCL5 shows strong neurotrophic properties. In this study, we found that CCL5 facilitates hippocampal glucoregulatory efficiency. CCL5 promotes aerobic glucose metabolism, mitochondrial ATP generation and purine synthesis, improving synaptic plasticity and enhancing memory and cognition functions.

## Introduction

Chemokines are pro-inflammatory cytokines with chemoattractant properties that have been described as important regulators of peripheral and central immune responses [1–3]. As such, they are particularly involved in the cross-talk between neurons and glial cells as well as neuroinflammatory components in neuropsychiatric diseases such as schizophrenia, mood disorders, and Alzheimer’s disease (AD) [4]. In addition to such roles in pathological conditions, chemokines are also thought to be important homeostatic regulators in the central nervous system [2, 3].

CCL5 (C–C motif chemokine ligand, also known as RANTES: Regulated–on–Activation–Normal–T–cell–Expressed–and-Secreted) is a chemokine known to favor chemotactic signaling to T lymphocytes, basophils, and eosinophils in the peripheral immune system. Higher levels of CCL5 have been associated with Alzheimer’s disease (AD) in patients and the ApoE genotype [5]; however, the link to pathology and plasticity has been little studied. CCL5 has also been shown to favor clearance of amyloid-β deposits by microglia and to improve memory function in an AD mouse model [6]. Other studies have also suggested a presumed beneficial role of CCL5 in AD [7, 8], underlying its complex role in pathological conditions.

CCL5 is constitutively expressed by microglia and astrocytes in the brain. Puzzlingly, recent in situ hybridization studies have revealed that about 80% of CCL5 mRNA is expressed by neurons in the hippocampus, amygdala, ventral tegmental area, and hypothalamus [9]. The physiological role of CCL5 remains however largely unknown. We previously demonstrated that CCL5 contributes to neuronal activity and neurite outgrowth in a Huntington’s Disease model, supporting that this chemokine may act as a trophic factor [10]. CCL5 has also been shown as a downstream moiety for hepatocyte growth factor (HGF) activity on axonal outgrowth [11].

Neurite outgrowth and synapse formation require intracellular structures, intracellular transport and as well as a precise location of energy supply [12]. Dendritic mitochondrial activity is critical for synaptic plasticity and it provides ATP at active synaptic terminals [12]; in addition, biogenesis of mitochondria through peroxisome proliferator-activated receptor gamma coactivator-1α (PGC1-α) is a critical response for new mitochondria generation to ensure energy for axonal growth and regeneration [13].

Glucose is the most important energy source for memory formation and studies have shown that glucose is a potential cognitive enhancer in both elderly and young people. Alzheimer’s disease, schizophrenia, mild head injury, and mild cognitive impairments all manifest glucoregulatory complications [14]. Cerebral insulin and brain extracellular glucose availability are the most important mediators of glucose memory enhancement effects. In addition to glucose, AMP-dependent protein kinase (AMPK), activated by the AMP/ATP ratio, senses changes in cellular energy status to increase glucose metabolism and mitochondrial respiration. Studies have demonstrated that AMPK is an essential element in the regulation of neuroenergetic metabolic plasticity during synaptic activation[15]; however, hyperactivation of neuronal AMPK could induce synapse loss by autophagy [16]. The balance of energy status in synapses upon AMPK activity may be thus critical for cognitive function. We recently demonstrated that the hypothalamic CCL5/CCR5 axis in glucose uptake and AMPK activity regulation contributes to systemic insulin sensitivity and glucose tolerance [17].

The mechanism of how CCL5 promotes neurite outgrowth remains unclear. CCL5 has many receptors including CCR1, CCR3, CCR5, and GPR75. However, most studies of CCL5 receptors have suggested a function in memory suppression. The activation of the CCR5 receptor inhibits hippocampal neuron long-term potentiation via its ligand MIP1-alpha/CCL3 and modulates MAPKs and CREB signaling [18, 19]; inhibiting CCR5 by Maraviroc prevents memory impairment in both stroke and TBI-induced memory loss [18, 20]. Treating animals with the CCR3 antagonist-YM344031 attenuates neurodegenerative pathologies and improves learning memory performance in an Alzheimer’s disease mouse model [21]. Thus, how CCL5 promotes neurite outgrowth and the nature of CCL5 cellular and system functions require further investigation.

Investigating the physiological function of CCL5, in the present study, we originally found that this chemokine physiologically regulates hippocampal synaptic plasticity, cognition, and spatial memory. We specifically demonstrate here that these effects are related to the ability of hippocampal CCL5 to promote aerobic energy metabolism and mitochondrial function, as shown by metabolomics and seahorse analysis, ultimately augmenting neuronal connectivity.

## Results

### Mice lacking CCL5 exhibit synapse loss, impaired neuronal activity, and memory dysfunction

**Fig. 1:**
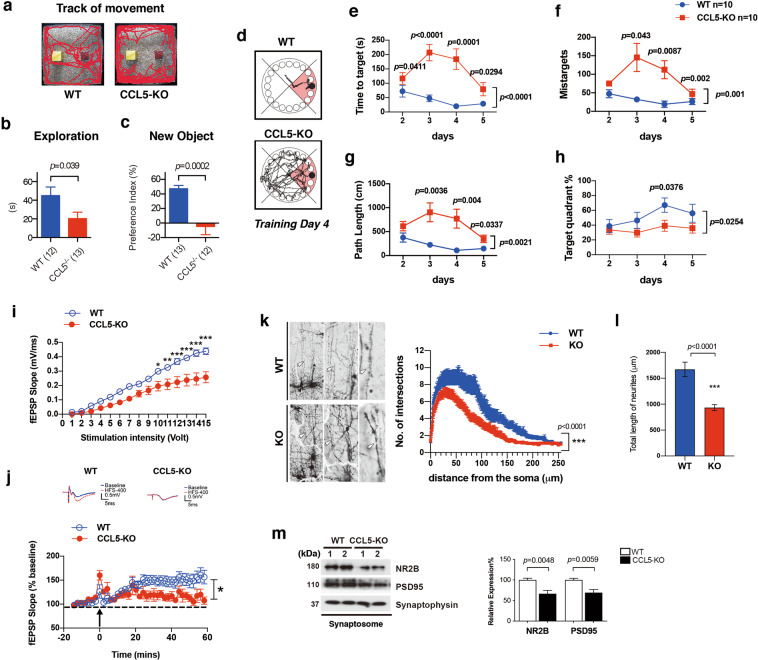
Memory, cognitive ability and hippocampal neuron activity are impaired in mice lacking CCL5. **a** The exploration tracks of WT and CCL5-KO mice in the box with objects in the novel object recognition (NOR) test. The exploration time (**b**) and preference for novel objects (**c**) were significantly lower in CCL5-KO mice. (Mann–Whitney test). **d** The movement track of WT and KO mice in the Barnes Maze (BM) on training day 4. The time to find target (**e**), missed target (**f**), and path length (**g**) were higher in CCL5-KO mice compared to age-matched WT mice; the time spent in target quadrant was less in CCL5-KO (**h**). (Day1 was the first day of training, which is not shown.) (Two-way ANOVA Bonferroni’s multiple comparisons test between two groups. Unpaired *t*-test was used to compare WT and KO at same day.) **i**, **j** Stimulation of hippocampal brain slices from both WT and CCL5-KO. The slope of fEPSPs was reduced in CCL5-KO compared to WT and marked reduction of LTP was found in CCL5-KO. (Two-Way ANOVA Bonferroni’s multiple comparison test, *p < 0.05; **p < 0.01; ***p < 0.001. WT: *n* = 10 slices of six mice, KO: *n* = 5 slices of two mice.). **k**, **l** Golgi staining identified neurite structure and spines; the neurite intersections and neurite length, analyzed by Sholl analysis, were reduced in KO mice. (Arrows point to the spines in WT and CCL5-KO mice) (**k**: Two-Way ANOVA, **l**: Unpaired *t*-test. WT: *n* = 14 of three mice, KO: *n* = 16 of three mice.) **m** Synaptic proteins −NR2B and PSD95 were reduced in hippocampal synaptosome fractions. Synaptophysin was used as a synaptosome control. (Unpaired *t*-test. *n* = 4–6.).

We first investigated the function of CCL5 in the brain using CCL5-knock out (CCL5-KO) mice. Behavioral investigations demonstrated that the learning memory performance of CCL5-KO mice in Barnes maze (BM) was not different from WT at 2 months of age (Supplementary Fig. 1a); the KO animals gradually showed impairments in learning and memory from 3 to 4 months of age (Fig. 1, Supplementary Fig. 1b). Most of the CCL5-KO mice showed memory impairment at age of 4 months. In the novel object recognition (NOR) task, the total exploration time and the preference for the new object were significantly lower in CCL5-KO mice (Fig. 1b, c, Fig. 1a shows the movement track of both groups of mice in the open field). Furthermore, using the Barnes maze, CCL5-KO mice demonstrated significant spatial memory impairments as exemplified by the enhanced time to target (Fig. 1e, d showed the movement track of WT and CCL5-KO mice at training day 4), number of mistargets (numbers of visited wrong holes. Fig. 1f), and path length (Fig. 1g). In addition, time spent in the target quadrant was significantly reduced during the training period (Fig. 1h). These data, therefore, support that CCL5-KO animals exhibit hippocampal-dependent memory impairments. The brain and hippocampus sizes of mice were measured by MRI which showed no difference between WT and CCL5-KO animals (Supplementary Fig. 1c, d). The distributions of neurons in the brain were labeled by Nissl staining. There were no significant differences in hippocampus DG, CA3, CA1, and medial prefrontal cortex regions between WT and KO mice (Supplementary Fig. 1e, f).

To correlate with these behavioral data, we analyzed hippocampal plasticity (LTP) at CA3-CA1 Schaffer collateral synapses in WT and CCL5-KO mice. We observed that CA1 fEPSP slope, upon collateral stimulation, was lower in CCL5-KO hippocampal slices as compared to WT animals (Fig. 1i). The addition of CCL5 into KO mouse brain slices showed a dose-dependent increase of CA1 fEPSPs (Supplementary Fig. 2a–c; CCL5: 50–500 pg/ml). In addition, significant suppression of LTP was found in CCL5-KO animals (Fig. 1j). LTP was also increased by CCL5; brain slices treated with higher dosages of CCL5 (250, 500 pg/ml) showed LTP as good as WT slices (Supplementary Fig. 2d). In accordance with such plasticity impairments, we also found, using Golgi staining and synaptosome purification, that neurite intersections and neurite length were significantly lower (Fig. 1k, l), while synaptosomal expression of both PSD95 and NR2B proteins were reduced (Fig. 1m) in CCL5-KO mice. Taken together, these data strongly suggest that CCL5 is involved in the maintenance of hippocampal synapse structure and function, supporting playing an important role in memory formation.

### CCL5 re-expression into CCL5-KO hippocampus improves memory performance and synaptic protein expression

**Fig. 2:**
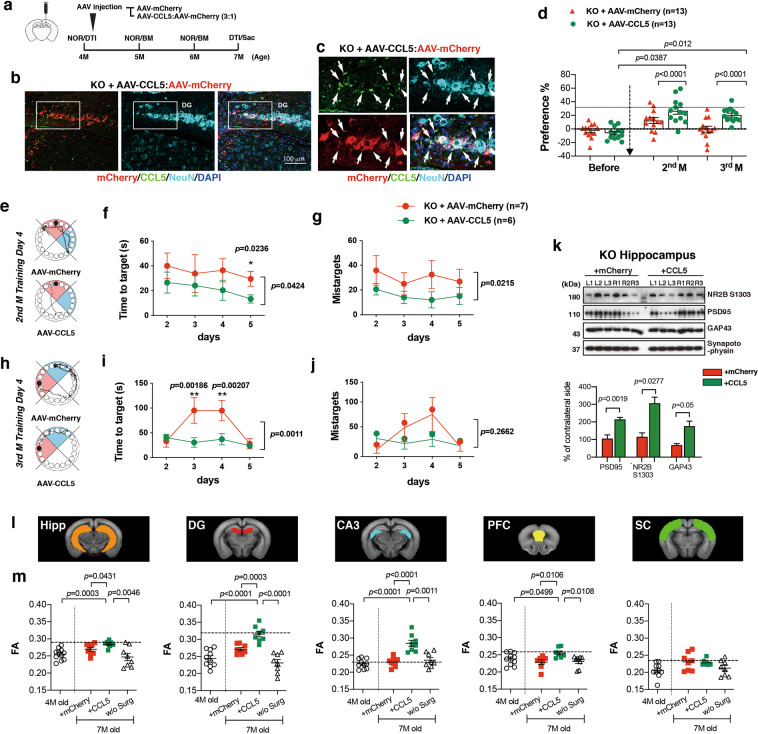
Reintroducing CCL5 into hippocampus improved memory performance, synapse-related protein expression, and neuron connectivity in CCL5-KO mice. **a** The scheme of experimental design. **b** The distributions of AAV-CCL5 and AAV-mCherry in mouse hippocampus. Hippocampal regional distributions and colocalization of CCL5 (green), mCherry (red), and neuron marker––NeuN (Cyan) are enlarged in (**c**). (Scale bar = 100 μm). **d** The preference for new objects increased in CCL5-KO mice receiving AAV-CCL5 after 2–3 months compared to mice receiving AAV-mCherry alone. (Paired *t*-test to compare mice before and after AAV injection. Mann–Whitney test for AAV-mCherry and AAV-CCL5 groups.) **e**, **h** The movement track of mice in the BM on training day 4. Target box labeled in black-circle in red quadrant and blue quadrant indicated the hidden box in the previous month. **f**, **i** The time to find the target in AAV-mCherry and AAV-CCL5 injected mice after 2 and 3 months; and (**g**, **j**) the mistarget number in both groups of mice. (Two-way ANOVA between groups. Unpaired *t*-test for single time point comparisons.) **k** The synaptic proteins phosphor-NR2B-S1303, PSD95, and GAP43 were increased in AAV-CCL5 injected mouse hippocampal synaptosome fractions. (Numbers indicate three separate mice; L: left and R: right hippocampus with AAV expression. Mann–Whitney test, *n* = 4–6.) **l** Color labels the regions of interest (ROI), including hippocampus (Hipp, Orange), dentate gyrus (DG, Red), CA3 (Cyan), medial prefrontal cortex (PFC, Yellow), and somatosensory cortex (SC, Green). **m** The FA values in Hipp, DG, CA3, and PFC regions were significantly increased in the AAV-CCL5 injected group compared to AAV-mCherry injected, age-matched mice without surgery (7-months old) and also before surgery (4-months old) groups (Dashed line show the average value in WT, in Fig. 5). (Mann–Whitney test.).

We next studied if re-expression of CCL5 in hippocampal neurons of 4 months old CCL5-KO mice could restore memory function and synaptic integrity. As shown in Fig. 2a, AAV-CCL5 or AAV-mCherry were administered into the right hippocampal dentate gyrus (DG) in 4-month-old mice and their impact studied 3 months later. The distribution and expression of CCL5 in CCL5-KO animals after administration of AAV-CCL5 were determined using a CCL5-specific antibody (Supplementary Fig. 3b, enlarged in b1, b2); AAV-mCherry was injected as control (Supplementary Fig. 3a). AAV was expressed in the DG area and adjacent hippocampal regions (Supplementary Fig. 3a, b). Expression was essentially neuronal since, as shown in Fig. 2b, c, mCherry was specifically expressed by neurons, as demonstrated by full NeuN co-labeling (Fig. 2b, c, and Supplementary Fig. 3c, d).

The impact of CCL5 re-expression on learning and memory performance was then examined using NOR and BM tasks, performed every month following AAV administration. The behavioral performance was not different during the first month after injection (Supplementary Fig. 3e). However, as shown in Fig. 2d, the preference of CCL5-KO mice for the new object (NOR) significantly increased in mice injected with AAV-CCL5 after 2–3 months, as compared to mice injected with the AAV-mCherry control. In addition, the time needed to find the target in Barnes Maze was reduced in the AAV-CCL5-treated animals vs. AAV-mCherry controls, 2–3 months post injection (Fig. 2e, h, f, i); the “mistarget” number was also found to be lower in the AAV-CCL5 group (Fig. 2g, j). The time spent in the target quadrant was higher in the AAV-CCL5 group by the 2nd month; however, the path length was not different between these two groups (Supplementary Fig. 3f, g). In line with improved memory, expressions of synaptic protein PSD 95, GAP43 and the phosphorylation of NR2B at serine 1303 (NR2B^S1303^) were increased on the AAV-CCL5 injected side in hippocampal synaptosome fractions (Fig. 2k).

In accordance with these observations, we observed that microstructural connectivity, assessed by diffusion-tensor image (DTI), was enhanced by CCL5 re-expression. Indeed, while fractional anisotropy (FA) values in CCL5-KO mouse whole hippocampus (orange region, Fig. 2l), dentate gyrus (DG; red), CA3 (cyan) to medial prefrontal cortex (yellow) were lower than in WT (dashed line indicates the mean values in WT), it was significantly higher in mice receiving AAV-CCL5 as compared to those receiving mCherry (Fig. 2m). Importantly, in those areas the FA values in CCL5 expressing groups reached values similar to WT (dashed line). The somatosensory cortex region (SC) which did not “connect” to the memory-cognition circuit showed no changes in the FA values after AAV-CCL5 expression (Fig. 2l, green, and Fig. 2m). The whole brain and hippocampus sizes were not increased by AAV-CCL5 expression in KO mice (Supplementary Fig. 1c, d). Also, the number of neurons in DG, CA3, CA1, and PFC regions was similar in KO mice expressing AAV-mCherry and AAV-CCL5 (Supplementary Fig. 1g, h). Altogether, these data indicate that re-expression of CCL5 in the DG of CCL5-KO mice improves hippocampal synaptology, linked to better cognition.

### CCL5 is critical for glucose metabolism in the hippocampus, mitochondrial function, and de novo purine synthesis

We previously showed that the CCL5-CCR5 axis regulates glucose uptake and insulin signaling activation in the hypothalamus [17]. Notably, we found that CCL5 promotes glucose transporter 4 (GLUT4) translocation to membrane and favors IRS-1 function. In order to evaluate if cognition and synaptic improvement induced by CCL5 were related to a change in hippocampal metabolism, we used LC Q-ToF MS to analyze metabolites from both WT versus CCL5-KO mouse hippocampus as well as KO animals with control AAV-mCherry versus AAV-CCL5-mediated overexpression. We detected significant metabolome changes in CCL5-KO vs. WT as well as in CCL5-KO mice re-expressing CCL5 vs animals injected with the AAV-mCherry control (Supplementary Fig. 4a, c, d, f–i). Notably, CCL5 overexpression in KO mouse hippocampus altered 30 related compounds, amongst which 28 overlapped with those 37 differentially changed in CCL5-KO mice vs. WT (Supplementary Fig. 4l). Our analysis thus supports that CCL5 re-expression in CCL5-KO mice reversed changes in metabolic pathways associated with CCL5 deletion. Moreover, eight compounds found overexpressed in the hippocampus of KO mice were downregulated following AAV-CCL5 injection whereas 20 compounds down-regulated in KO mice were upregulated in KO mice injected with AAV-CCL5. Among these pathways, our results show that CCL5 modulates five metabolic pathways, involved in glucose metabolism namely (1) fructose and mannose degradation, (2) glycolysis, (3) glutamate metabolism, (4) purine metabolism, and (5) the gluconeogenesis pathway (Supplementary Fig. 4m).

**Fig. 3:**
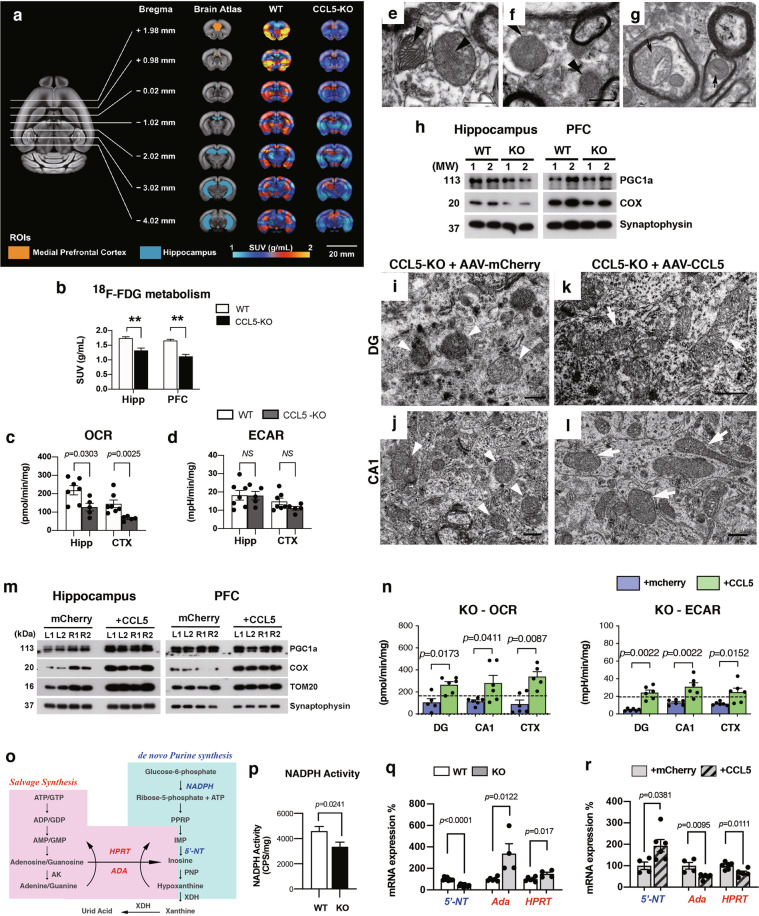
CCL5 participates in brain glucose aerobic metabolism and mitochondrial functions. **a** MicroPET images averaged over all mice from both WT and CCL5-KO, and the average regional [18 F] FDG uptake is presented as standardized uptake value (SUV). Regions of interest (ROIs), defined on a series of coronal sections of the co-registered PET-MRI image of the mouse brain, were used to determine [18 F] FDG uptake in bilateral medial prefrontal cortex (PFC) and hippocampus (Hipp). Precise localization of ROIs was aligned on the brain atlas to the matching structures on the brain images (PFC-orange; Hipp-blue). **b** PET images acquired from WT and CCL5-KO mice were compared. (** indicates *p* < 0.01, Kruskal–Wallis test compared with the WT group. *n* = 4 for each group.) The energy utilization rate for oxygen consumption (OCR) (**c**) and glycolysis - extracellular acidification rate (ECAR) (**d**) in WT and KO hippocampus and cortex regions were compared. (Mann–Whitney test, *n* = 6–8. NS: No significant difference.) **e**–**g** Electron microscopic images of mitochondrial structure revealed outer membrane abnormalities in KO mouse brains (**f**, arrowheads) and loss of inner cristae (arrows in **g**) compared to normal mitochondria in WT (**e**, arrowheads) (Scale bar = 5 μm). **h** Levels of mitochondrial proteins––COX and mitochondria biogenesis protein––PGC1α in CCL5-KO hippocampus synaptosome fractions but not in prefrontal cortex were lower than in WT. (Mann–Whitney test, *n* = 5–7. Numbers indicated independent mice.) **i**–**l** The ultrastructure of mitochondria in mouse hippocampal DG and CA1 regions after AAV-mCherry and AAV-CCL5 expression studied with electron microscopy. The outer membrane and mitochondrial size were increased in KO mice receiving AAV-CCL5 (**k**–**l**: Arrows point to the elongated mitochondria and dense inner cristae) compared to mice receiving AAV-mCherry (**i**–**j**: Arrowheads point to the loss of outer membrane and inner cristae.) (2–3 mice were analyzed and 20 pictures were taken for each brain area). **m** The mitochondrial proteins COX and TOM20 were increased on both sides of AAV-CCL5 injected mice in hippocampal and PFC synaptosome fractions. PGC1-α was increased in AAV-CCL5 expressing hippocampus but not in PFC. (Numbers indicated separate mice; L: left and R: right hippocampus with AAV. Mann–Whitney test, *n* = 4–6.) **n** The respiratory activity of mitochondria in mouse brain tissues was measured after mice were injected with AAV-mCherry or AAV-CCL5 for 3 months. The OCR and ECAR increased in AAV-CCL5 expressing hippocampal DG, CA1, and cortex regions. (Dashed line point to the average value in WT-mCherry in Fig. 5; Mann–Whitney test, *n* = 6.) **o** The diagram of pentose phosphate pathway (PPP) and purine metabolism-related molecules and enzymes. **p** The key enzyme for PPP––NADPH activity in WT and KO mice hippocampus. (Mann–Whitney test, *n* = 10.) **q** Quantitative PCR analysis of the de novo purine metabolism enzyme 5’-neucleotide (5’-NT) and salvage purine synthesis enzyme adenosine deaminase (Ada) and hypoxanthine-guanine phosphoribosyl transferase (HPRT) between WT and KO mice or CCL5-KO mice receiving AAV-CCL5 or mCherry (**r**). (Data was normalized to contralateral side and analyzed by unpaired *t*-test.).

Changes in glucose uptake in the mouse brain were validated using ^18^FDG (Fluorodeoxyglucose) positron emission tomography (PET) analysis. KO mice showed a significantly lower signal intensity as compared to WT mice in the hippocampal and medial prefrontal cortex (PFC) regions (Fig. 3a, b), supporting the impaired hippocampal glucose metabolism pathway of CCL5-KO suggested by the metabolome approach above. In line with this, the oxygen consumption rate (OCR) was found significantly lower in CCL5-KO vs. WT animals using the seahorse analyzer (Fig. 3c) without alteration of glycolysis activity (ECAR; Fig. 3d) in both hippocampus and cortex regions. Together, our data suggests that lack of hippocampal CCL5 impairs aerobic glucose metabolism, presumably involving a reduced mitochondrial oxidative activity. In our protein blot analysis, the energy deficiency shown as AMPKα phosphorylation T172 (pAMPKα) was not different between WT and KO hippocampus. However, insulin resistance shows that increased phosphorylated insulin response substract-1 S302 (pIRS-1 S302) and that reduced phosphorylated Akt S473 (pAkt S473), was found in KO mice (Supplementary Fig. 5a, b).

Aerobic glucose metabolism requires mitochondrial activity to generate increased levels of ATP via the Krebs cycle. We next examined brain mitochondria using electron microscopy in order to characterize the effect of CCL5 loss on mitochondrial structure. Structural abnormalities such as mitochondria without outer membranes (Fig. 3f) and with less inner membrane cristae (Fig. 3g), were found in CCL5-KO mouse hippocampus compared to WT animals (Fig. 3e). Mitochondrial structural integrity was restored by re-expression in CCL5-KO mice. The AAV-mCherry-injected CCL5-KO mice showed missing outer membranes and fewer mitochondrial cristae in DG (injection area, Fig. 3i), CA1 (Fig. 3j), and sensory cortex regions (Supplementary Fig. 6a); the outer membranes and the length of mitochondria were increased in AAV-CCL5 injected KO animals (Arrows in Fig. 3k, l) but not in regions such as sensory cortex, where CCL5 had not been re-expressed (Supplementary Fig. 6b). Furthermore, in synaptosomal hippocampal fractions, CCL5-KO mice exhibited a significantly lower expression of cytochrome c oxidase (COX) which is involved in respiration as well as of peroxisome proliferator-activated receptor gamma coactivator 1-alpha (PGC1α), which is involved in mitochondrial biogenesis; the level of COX, but not PGC1α, was also lower in the PFC synaptosome fraction (Fig. 3h, Supplementary Fig. 7a). In line with this, the levels of mitochondrial proteins COX, TOM20, and PGC-1α but not PGC-1α in PFC were increased in KO mice hippocampus re-expressing neuronal CCL5 (Fig. 3m, Supplementary Fig. 7b, c). Importantly, OCR and ECAR were significantly enhanced following AAV-CCL5 neuronal re-expression in the CCL5-KO mice (Fig. 3n, dashed line labels the mean value in WT), further supporting the functional involvement of CCL5. The synapse-related proteins––PSD95 and GAP43 in the PFC synaptosome fraction were not been affected which suggests CCL5 regulating mitochondria energy supply is critical for hippocampal neurons specifically (Supplementary Fig. 7d). In addition, hippocampal tissue energy status was enhanced in KO mice receiving AAV-CCL5, manifested as reduced pAMPKα expression; in addition, insulin signaling pathway activity was increased shown as reduced pIRS-1 S302 and increased pAkt S473 and pAkt T308 (Supplementary Fig. 5c, d).

To further validate the specificity of CCL5’s effect on hippocampal neurons or astrocytes, hippocampal neurons and astrocytes were cultured from WT mouse embryos and postnatal pups. An increased OCR and EACR were found in neurons treated with CCL5 in a dose-dependent fashion (Supplementary Fig. 6c, d). CCL5 treatment did not alter astrocytic OCR (Supplementary Fig. 6e) but inhibited ECAR (Supplementary Fig. 6f). Immunostaining with NeuN, GFAP, Iba-1, and CD11b in the primary cultures showed the purity of the primary neuron and astrocyte cultures (Supplementary Fig. 6g–k). Together, this data supports that CCL5 increases hippocampal neuron aerobic metabolism.

Mitochondria not only provide the ATP supply and oxidative respiration in cells but are also important for purinosome––nucleotide purine metabolism (Fig. 3o). The regulatory enzyme activity for the pentose phosphate pathway (PPP), NADPH, was significantly lower in CCL5-KO brain tissue (Fig. 3p). The mRNA level of 5ʹ-nucleotidases in de novo purine synthesis (5ʹ-NT) was reduced whereas the salvage purine synthesis enzyme––adenosine deaminase (ADA) and hypoxanthine-guanine phosphoribosyl transferase (HPRT) were increased (Fig. 3q) in CCL5-KO hippocampal tissue. These changes were reversed following CCL5 re-expression in CCL5-KO mice (Fig. 3r). These functional evaluations are in accordance with our metabolomic results and further support that hippocampal CCL5 is critical for neuronal glucose aerobic metabolism, mitochondrial biogenesis and mitochondrial activity for purine and ATP synthesis.

### Role of CCL5 in cellular energy status and synapse formation

**Fig. 4:**
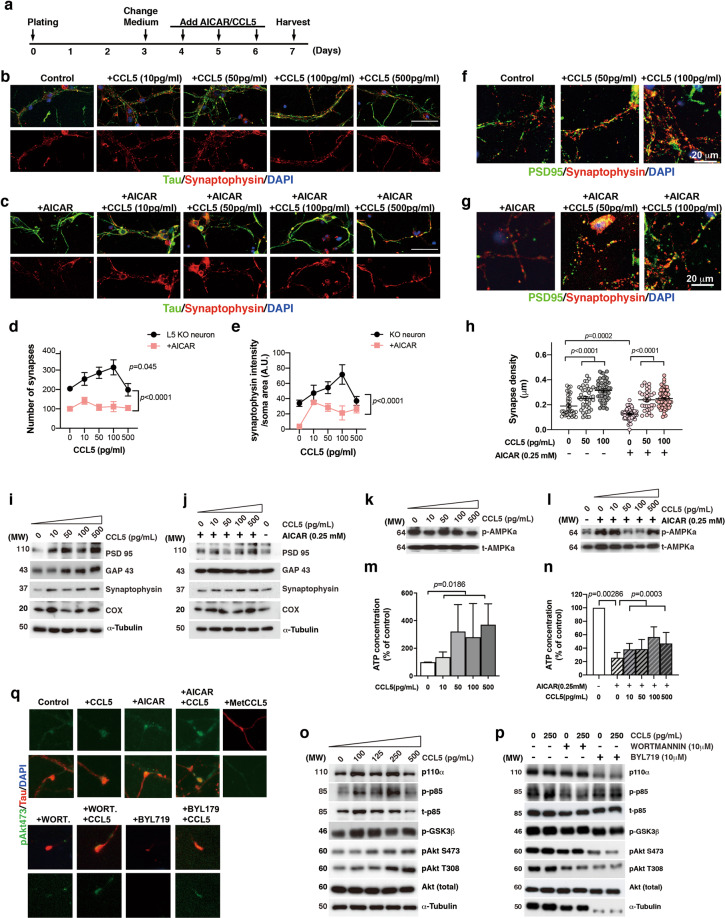
CCL5 activation of the PI3K/Akt pathway promotes synaptogenesis and ATP generation in hippocampal neurons. **a** The schematic of CCL5 and AICAR treatments in cultured hippocampal neurons. Hippocampal neurons were treated with CCL5 (0, 10, 50, 100, 500 pg/ml) and AICAR (25 mM) after 4 days in culture for 3 days. **b** Synapses labeled by synaptophysin (red) in hippocampus neurons (Tau in green), which was increased by CCL5 treatment. **c** AICAR treatment reduced synaptic structures; CCL5 treatment blocked AICAR-induced synapse reduction. (DAPI labeled nuclei in blue, Scale bar = 50 μm). The quantification of synapse number––synaptophysin (**d**) and intensity (**e**) under CCL5 and AICAR treatments. (One-way ANOVA). The co-localization of pre-synaptic protein––synaptophysin and post-synaptic protein––PSD95 upon CCL5 (**f**), and AICAR alone, or AICAR plus CCL5 (**g**). **h** The quantification of synaptic complex density upon CCL5 and AICAR (Mann–Whitney test for AICAR treatment and one-way ANOVA for CCL5 treatment). **i**–**j** The protein levels of synaptic proteins PSD95, GAP43, and synaptophysin and the mitochondrial protein––COX with CCL5 administration (**i**) and AICAR plus CCL5 (**j**). (**k**, **l**) CCL5 reduced the elevation of phosphor-AMPKα by AICAR and increased cellular ATP levels (**m**, **n**) in primary hippocampal neurons. (**m**–**n**: one-way ANOVA; n: unpaired *t*-test, AICAR compared to control. *n* = 4–6.) (**o**) CCL5 treatment activated PI3 Kinase signaling molecules––p110α, p85-p55, GSK3β to Akt in a dose dependent matter. **p** Pan-PI3K inhibitor––Wortmannin and P110α specific inhibitor––BYL179 blocked CCL5 induced PI3K pathway activation. **q** Synapse localization of Akt S473 (green) under normal neuron basal medium––control compared to treatments with CCL5, AICAR, the CCL5 antagonist––MetCCL5, the PI3K inhibitor––Wortmannin, or BYL179 in primary hippocampal neurons. Tau (red) labeled neuritis.

We further investigated the potential role of CCL5 in cellular energy status and synapse formation using primary hippocampal neurons in vitro. Specifically, we used primary hippocampal neurons from CCL5-KO mice to avoid the confounding effects of endogenous CCL5. Neurons were cultured for 3 days after which different doses of exogenous CCL5 (0, 10, 50, 100, 500 pg/ml) were administered; AICAR (0.25 mM) was used to increase cellular AMPKα phosphorylation, in order to induce energy deficiency (Fig. 4a). Neurons cultured with CCL5 showed more synaptic structures evidenced by immunochemical staining for synaptophysin (Fig. 4b-red, Supplementary Fig. 8a, Fig. 4d, e); synaptic proteins such as GAP43, PSD95, synaptophysin, and mitochondrial––COX were also increased as shown by protein blot (Fig. 4i, Supplementary Fig. 8e–h). The synaptic structure complex shown by colocalization of synaptophysin (pre-synaptic protein, red) and PSD95 (post-synaptic protein, green) was also increased by CCL5 treatment (Fig. 4f, h; Supplementary Fig. 8c). Labeling of synaptophysin in neurons was reduced after treatment with AICAR (Fig. 4c–e). Co-treatment with CCL5 after 30 min of AICAR exposure restored synaptic structure (Fig. 4c–e) as well as both mitochondrial and synaptic proteins (Fig. 4j, Supplementary Fig. 8b, i–l). The synaptic complex was also reduced by AICAR treatment and increased with CCL5 (Fig. 4g, h; Supplementary Fig. 8d).

Since AICAR increased phosphorylation of AMPKα, we further studied if CCL5 could change cellular ATP status using both an ATP assay and cellular p-AMPKα levels. Increased cellular ATP was found in CCL5-treated primary hippocampal neurons together with reduced p-AMPKα (Fig. 4k, m, Supplementary Fig. 8m). AICAR treatment increased cellular p-AMPKα and reduced cellular ATP; CCL5 co-treatment with AICAR eliminated the increased p-AMPKα upon AICAR exposure and restored cellular ATP levels (Fig. 4l, n, Supplementary Fig. 8n).

The PI3K/GSK3β/Akt pathway is an important signaling pathway promoting axonal and neurite outgrowth. Energy deficiency or AICAR treatment blocks PI3K binding to kinesin light chain (KLC) and axonal tip localization of pAkt, thus reducing axon outgrowth [22]. CCL5 treatment enhances axon-dendrite differentiation and neurite elongation during neuron development. We found that AICAR treatment blocked axon-dendrite differentiation and neurite elongation which could be successfully reversed by CCL5 (Supplementary Fig. 9a–d). CCL5 treatment also increased p110α, and the phosphorylation of p85, GSK3β, and Akt signaling in primary hippocampal neurons in a dose-dependent fashion (Fig. 4o); this signal could be blocked by the p110α specific blocker––BYL719 (10 μM) and the PI3K inhibitor––WORTMANNIN (10 μM) (Fig. 4p). CCL5 also promoted pAkt movement to the neurite terminal––synapse structure; both AICAR and the CCL5 antagonist-^Met^CCL5 treatment reduced pAkt synapse localization. CCL5 also reversed the inhibitory effect of pAkt synapse translocation after BYL719 and WORTMANNIN exposure (Fig. 4q, Supplementary Fig. 9e–m).

Our data, therefore, demonstrate that CCL5 increases cellular energy and restores synapses lost because of cellular energy deficiency. The CCL5 enhancement is through activating the PI3K-p110α/GSK3β/Akt pathway and augmenting pAkt synaptic localization.

### Overexpressing CCL5 in hippocampus promotes aerobic glucose metabolism, purine synthesis, synapse formation, and enhances learning and mnemonic activity in WT animals

AAV-CCL5 and AAV-mCherry were administered into the right hippocampal region of 4-months-old WT mice to further validate the importance of CCL5 in aerobic glucose metabolism for memory. The performance in learning and memory was examined by NOR and BM tests. The experimental design is shown in Supplementary Fig. 10a. The expressions of mCherry (red), as well as CCL5 overexpressed through AAV, and endogenous CCL5 were labeled by a CCL5 specific antibody in mouse brains (Supplementary Fig. 10b, c, green).

**Fig. 5:**
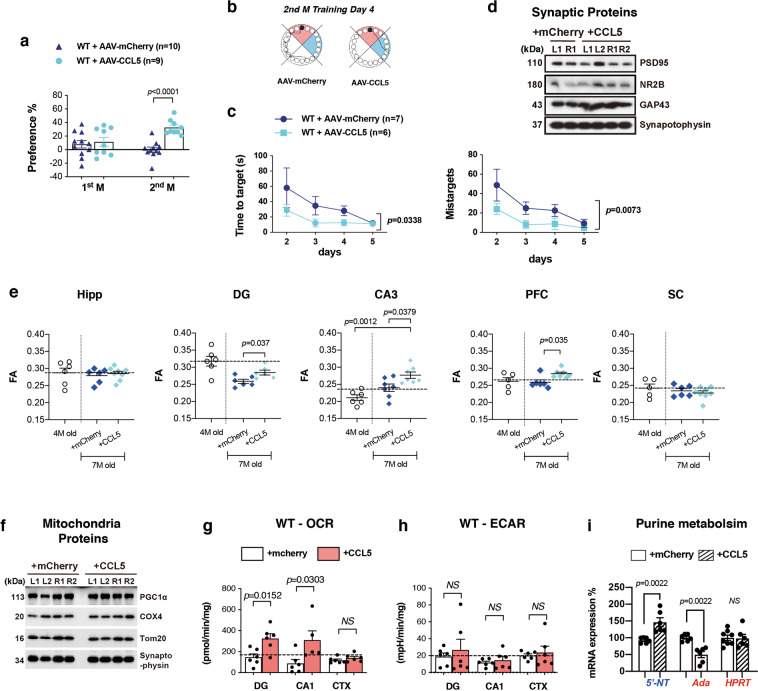
Expressing CCL5 in WT mouse hippocampus enhanced mouse memory performance. **a** The preference for a new object in the NOR test was increased in AAV-CCL5 injected WT mice after 2 months. (Mann–Whitney test). **b** The movement track of mice in the Barnes Maze (BM) on training day 4 in AAV-mCherry and AAV-CCL5 injected mice. (Target box labeled in black-circle in red quadrant and blue quadrant indicated the hidden box in last month.) **c** Mice spent less time in finding targets and made fewer mistakes, mistargets in the BM task, 2 months after receiving AAV-CCL5. (Two-ways ANOVA). **d** The levels of neuron plasticity related proteins NR2B and GAP43 in mouse hippocampus synaptosome fractions were increased in AAV-CCL5 injected right (R) hemisphere but not PSD95, (**f**) PGC1-α and COX. (Numbers indicate independent mouse. R: right, injection side, L: left, contralateral side.) **e** The in vivo structural changes were evaluated by MRI-DTI. The FA value in DG, CA3, and PFC significantly increased in AAV-CCL5 injected WT mice after 3 months compared to AAV-mCherry injected mice, but not in whole hippocampus and somatosensory cortex (SC). (Mann–Whitney test, *n* = 6–7.) (**g**, **h**) The respiratory activity in OCR increased in the hippocampal DG and CA1 in WT mice but not ECAR-glycolysis. (Mann–Whitney test, *n* = 6.) (**i**) AAV-CCL5 administration increased *5ʹ-NT* gene expression in WT mouse hippocampus and reduced *Ada* gene expression but *HPRT* was not changed. (Mann–Whitney test, *n* = 4–6.NS: No significant difference.).

Memory performance was not different between control AAV-mCherry and AAV-CCL5 groups during the first month as measured by NOR (Fig. 5a) and BM performance (Supplementary Fig. 10d) similar to KO mice. However, WT mice receiving AAV-CCL5 showed enhanced memory performance after 2 months. In fact, the preference for a new object was increased after 2-months expression of AAV-CCL5 (Fig. 5a). Also, the time for finding targets and mistarget numbers were reduced in BM trials (Fig. 5b, c). In addition, mice receiving AAV-CCL5 spent more time in the target quadrant and had less path length to find the target (Supplementary Fig. 10e). In line with this, plasticity related proteins such as NR2B was increased in WT mice hippocampus synaptosome fraction (Fig. 5d, Supplementary Fig. 7e). Further, DTI-FA values were found to be increased in the DG, CA3 to PFC of AAV-CCL5 injected WT mice (Fig. 5e), but not increased in whole hippocampus and somatosensory cortex (SC). The size of whole brain and hippocampus were not changed by AAV-CCL5 expression (Supplementary Fig. 1c, d). Altogether these data suggest an increased microstructural connectivity in CCL5 overexpressing WT mice.

Finally, we also used LC Q-ToF MS to analyze hippocampal metabolites from WT animals injected with control AAV-mCherry or AAV-CCL5, and compared pathways to those altered in CCL5-KO vs WT (Supplementary Fig. 4b, e, j, k). CCL5 overexpressing hippocampal tissues showed an alteration of 20 identified pathway-related compounds; among these, 18 pathways showed compounds that overlapped WT versus KO and 12 pathways showed compounds that overlapped in three comparisons which specifically involve glutamate and purine metabolism (Supplementary Fig. 4l, m, gray marked in m.). Aerobic metabolism - OCR in CA1 and DG regions, but not in cortex region, was increased after expression of AAV-CCL5 (Fig. 5g). The ECAR was not different after CCL5 treatment in DG, CA1, and cortex regions (Fig. 5h). Mitochondrial proteins – PGC1-α and COX were not different after treatment with CCL5 (Fig. 5f, Supplementary Fig. 7f, g). The pAMPKα level was not different in WT mice receiving AAV-CCL5; interestingly, insulin signaling pathway activity was enhanced, manifested as reducing pIRS-1 S302 and increasing pIRS-1T612, pAkt T308 and pAkt S473 in AAV-CCL5 injected mouse hippocampus. (Supplementary Fig. 5e, f). The purine metabolism enzyme––5’-NT was increased and ADA was reduced by overexpression of AAV-CCL5 in WT mouse hippocampus, but *HPRT* was not affected (Fig. 5i). These findings confirm metabolomic results, further supporting that CCL5 is involved in hippocampal neuron glucose metabolism and ATP generation.

## Discussion

The present study identifies a critical role of the chemokine CCL5 in hippocampal-dependent memory processes. Our data also specifically reveal, for the first time, that CCL5 by promoting neuronal glucose uptake and ATP generation by neuronal mitochondria, sustains neuronal activity and connectivity.

Mice lacking CCL5 showed impaired performance in both recognition memory and spatial memory at about 4 months of age, which was reversed by chemokine re-expression in the hippocampus. CCL5 is expressed constitutively in both developing and mature retina and is important for inner retina maturation and visual function [23]. A recent study showed that CCL5 deficiency altered the phenotype of rod bipolar cells and inner retinal circuitry in murine retina [24]. Since, both NOR and BM tasks require mice to visualize spatial cues, the learning and memory performance impairments seen in CCL5-KO mice might thus be related to the function of CCL5 in the retina. However, CCL5 administration in WT animals enhanced cognition and learning/memory in WT mice i.e., with normal visual function. Moreover, CCL5 expression in WT and KO mice improved neuronal activity as measured by diffusion MRI, which is important for proper memory function. It is therefore unlikely that memory regulation by CCL5 is mediated though retinal-dependent processes. Rather, the Golgi staining, synaptosome protein analyses and electrophysiological measurement taken together support that behavioral impairments seen in CCL5-KO mice result from the loss of synaptic integrity and function. This was further supported by recovery obtained following neuronal re-expression of AAV-CCL5.

CCL5 has been recently shown to be expressed in neurons of the ventral tegmental area (VTA), nucleus accumbens, striatum, prefrontal cortex (PFC), and hippocampus [9]. Here, in addition to synaptic changes, we observed that the neuron connectivity, measured by FA, values increased after expression of CCL5 in the hippocampus. This suggests that CCL5 might contribute to the development of functional connectivity between hippocampus and other brain regions. Other studies also emphasize that CCL5 regulates both glutamatergic neurotransmission [25] and glutamate metabolism [26, 27], important for learning and memory [28]. In line with this, our metabolomic data support that hippocampal glutamate dynamics might be affected both in CCL5 KO mice and following AAV-CCL5 intracerebral injection. This may explain our electrophysiological changes, observed under both basal and high-frequency electrical stimulation. Finally, our in vitro data also support that CCL5 might be involved in synapse remodeling. Microglia are an important effector of synaptic pruning and remodeling [29] not only during ageing and neurodegeneration but also during neurodevelopment. Depletion or inhibition of microglia prevent forgetting and the dissociation of engram cells [30]. As CCL5 is a strong chemokine for activating microglia [6, 31, 32], it is conceivable that neuronal CCL5 regulates synaptic pruning by microglia, contributing to plasticity and memory formation.

We analyzed the activation of astrocytes using GFAP and microglia-Iba-1 in WT and KO mice after AAV expression. We found little change in GFAP activation between different mouse groups (Supplementary Fig. 11d, e); but mouse without CCL5 and overexpressing CCL5 in KO mice have more Iba-1 positive cells (Supplementary Fig. 11f, g). The number of Iba-1 or GFAP positive cells was not changed in WT animals expressing AAV-CCL5. The pro-inflammatory chemokines as TNF-α and IL-1ß were not changed also (Supplementary Fig. 11a–c); however, IL-10 was significantly increased when overexpressing AAV-CCL5 in KO mouse brain (Supplementary Fig. 11b). This suggests the increased Iba-1 positive cells in AAV-CCL5 expressing KO mice might belong to type 2 microglia, but which receptor mediates this process remains unclear. We think 4-month old CCL5-KO mice progressively degeneration thus induced microglia activation; whenever overexpressing AAV-CCL5 3 months might change the characters and functions of microglia. The CCL5-CCR5 axis has been thought to be involved in the inflammation-related memory impairment seen after brain injury. Inhibiting CCR5 function by Maraviroc improves memory impairment after stoke and traumatic brain injury [18, 20]. However in our study, we found lower CCR5 receptor expression in the KO mouse brain (Supplementary Fig. 12a); interestingly, lower CCR5 expression was also found in both KO and WT mouse hippocampus when re-expressing AAV-CCL5 (Supplementary Fig. 12b, c). In contrast, GPR75 and CCR3 were increased in AAV-CCL5 KO mice hippocampus (Supplementary Fig. 12b). GPR75 activates Gq signaling and promotes the activation of Akt, glycogen synthase kinase 3 beta (GSK3β), and extracellular signal-regulated kinase (Erk), all of which are important signaling pathways during memory formation [33]. In addition, GPR75 also participates in insulin secretion in pancreatic islets and improves glucose homeostasis [34]. These data suggest that what we found in the current study showing CCL5 promotion of bioenergy metabolism and synapse formation might go through GPR75. CCL5 might have different functions in synaptic and memory processes through different receptors. Most of these studies were done under pathological conditions such as brain trauma, stroke, or aged Alzheimer’s disease mouse models. The level of CCL5 would be much higher than under normal conditions (150–500 pg); also, other immune responses would be activated and chemokines would be released with chronic inflammation. We found that neuritic outgrowth, synaptic complex upregulation, and signaling molecule activation reached a plateau of about 500 pg/ml CCL5 (Fig. 4). CCL5 levels higher than 500 pg/ml did not support neurite outgrowth and synapse formation; this might explain why the inhibitors such as Maraviroc and YM344031 can reduce brain damage and memory dysfunction. The binding to different receptors could also determinate the function of CCL5.

Metabolomic analysis in particular demonstrated the impact of CCL5 on glucose and purine metabolism, favoring neuronal mitochondrial respiration and ATP generation. Energy regulation, especially glucose metabolism, is critical for memory formation and cognitive function [35], through mitochondria-based energy supply to synaptic function [36–38]. Many studies in humans and mouse models found that AD and diabetes share impaired brain glucose uptake and metabolism underlying cognitive dysfunctions [39–41]. Interestingly, cognitive disorders found in the AD brain were associated with brain insulin resistance linked to increased serine phosphorylation of IRS-1 [42]. We previously found that CCL5 was able to regulate insulin signaling through PI3 Kinase and Akt pathways, to increase glucose uptake and reduce the phosphorylation of AMPKα and IRS-1^S302^ [17]. This raises the possibility that this chemokine might exert positive effects on hippocampal function through regulation of glucose utilization and, potentially, mitochondrial functions. In this context, the present study provides new insights into how CCL5 regulates glucose utilization efficiency and contributes to memory-cognition function and synaptic integrity. Brain glucose uptake, indicated by PET and glucose metabolism, was found altered in the brain of CCL5-KO mice and CCL5 treatment increased aerobic metabolism, improved cellular ATP levels, and mitochondrial function both in vitro and in vivo. This may underlie improved synaptic status.

Lastly, our study also identified that CCL5 acts on purine metabolism. Purines are a group of molecules used by cells for many essential biochemical processes; involving adenosine triphosphate (ATP) for cellular energy and energy-dependent reactions, guanosine triphosphate (GTP) as building blocks for DNA and RNA synthesis, and nicotinamide adenine dinucleotide (NAD) and flavin adenine dinucleotide (FAD) as cofactors for cellular biochemical reactions. The cyclic nucleotides, cAMP and cGMP, are important key molecules and second messengers in many intracellular signaling pathways. Purines also serve other important roles in the nervous system termed the “purinergic system”. For example, adenosine acts as a neurotransmitter or trophic agent in the nervous system; in addition, extracellular nucleotides also can bind with ligand-gated P2X channels or G protein-coupled P2Y receptors on glia cells [43]. Purine metabolism pathways are related to neuronal diseases and neurodevelopment. Neurogenesis and gliogenesis initiated by purinergic signaling in the embryonic brain continue during postnatal development. Database studies using data published in PubMed, ScienceDirect, Scopus, and Web of Science identified that alanine, aspartate, glutamate, and purine metabolism might act as alternative pathways to overcome inadequate glucose and energy supply in neurodegeneration as AD and PD [44]. Purine metabolic enzymes and purinosome complex are located in mitochondria [45]. Lower NADPH activity and increased salvage purine metabolism were observed in CCL5-deficient mice whereas re-expression of CCL5 enhanced *5’-NT* mRNA and reduced *ADA* and *HPRT*. In addition, glutamate and purine metabolism were two major pathways enhanced by re-expressing CCL5 in the WT animals. Our observations correlate with recent studies showing deregulation of purine metabolism, manifested (1) as reduced *5’-NT* activity, (2) as increased *ADA* in parietal (PC) and temporal cortex (TC), and (3) as reduced ATP generation, and compromised mitochondria, in both AD patients or AD mice [46–48]. CCL5 treatment not only increased synaptic structures but also rescued reduced synaptic proteins due to energy shortage because of phospho-AMPKα by AICAR treatment. This supports the finding of impaired glucose metabolism and insulin signaling activation from lack of CCL5, and also shows the importance of CCL5 in neuronal energy supply. Energy supply is critical for neuronal function and synaptic activation. Nervous system tissue requires large amount of ATP to provide energy for trans membrane active pumps––Na+/K+ ATPase, and to sustain synaptic transmission.

Studies about the role of CCL5/RANTES in neurogenesis are limited. One study investigated the gene expression profile changed by CCL5/RANTES in the human neuron cell line––NT2-N [49]; cDNA microarrays identified a significant number of enzymes, transcription factors, and miscellaneous molecules involved in neuronal survival and differentiation, including neurite outgrowth and synaptogenesis. This study found cyclin-dependent kinase 5 regulatory subunits 2 (p39), cytochrome P450, Krüppel-like factor 7 (KLF-7), CBP/P300-interacting trans-activator 2, and BMP4 were upregulated upon CCL5/RANTES; in contrast, apoptosis regulators––BCL2-antagonist/killer1, and BCL2-asssociated athanogene 4 were downregulated after CCL5/RANTES treatment. P39 is a cofactor for CDK5 and responsible for increasing Cdk5 activity, actin dynamics, and dendritic morphogenesis [50, 51]. CDK5 and P39 signaling contribute to neuronal network formation, synaptic plasticity, and memory-cognition function [52]; they are especially associated with early-onset Alzheimer’s disease [53]. KLF-7 is a transcriptional activator that promotes axonal regeneration in the adult corticospinal tract [54] and dopaminergic neuron development in the olfactory bulb [55]. CCL5 also influences blood cell differentiation and development [56, 57]. Together, these studies suggest CCL5 might regulate differentiation-related factors to promote neurogenesis, synapse plasticity, and memory-cognition function in neurons and prevent neuronal apoptosis. However, the detailed mechanisms and pathways for CCL5 function in neurogenesis and development still require further investigation. In the current study, we did not see significant cell death in the 4-month-old CCL5-KO mouse hippocampus with both Caspase 3 and 9 protein levels and immunostaining of ApopTaq (Supplementary Fig. 13a, b, and i-j. Supplementary Fig. 13g shows data from a stroke mouse brain taken as positive control and Supplementary Fig. 13h shows a negative control). Expressing AAV-mCherry or AAV-CCL5 also did not influence cell death in both KO and WT mice (Supplementary Fig. 13c–f, and k-n).

We thus propose CCL5 has two functions to sustain hippocampus memory function. First, CCL5 can directly influence fEPSPs in the hippocampal brain slices; second, CCL5 can provide increased energy production and facilitate purine metabolism in neurons for synaptic and memory formation. These two together maintain hippocampal neuron functions during learning-memory processes. In conclusion, our results also support that CCL5 is critical for glucose metabolism and utilization in hippocampus, which contributes to neuronal synaptic activity and memory formation. In a historical context, CCL5 may be considered a critical part of the molecular substrate for the Hebbian synaptic changes underlying memory.

## Materials and methods

The detailed description of the materials and methods is provided in Supplementary Information.

### In vivo animal studies

C57BL/6 mice obtained from the National Laboratory Animal Center were used as wild type (WT). The CCL5^−/−^ (CCL5 knockout, CCL5-KO, B6.129P2-Ccl5^tm1Hso^/J, Stock No: 005090) were obtained from Jackson Laboratory. Only male mice and less than four generations were used in the current study. The mating and genotyping followed Jackson Laboratory protocols. Mice that are homozygous for the targeted mutation are viable, fertile, and the body size is normal; they do not display any gross physical or behavioral abnormalities according to both Jackson Laboratory descriptions and our own observations. However, the immune system shows delayed-type hyperactivation (DTH); the infiltrated macrophage number is reduced when mice are sensitized with subcutaneous injection of keyhole limpet hemocyanin or bovine purified protein derivatived from Jackson Laboratory descriptions.

Mice were housed in groups of 3–5 per cage with free access to food and water on a 12:12-h light/dark cycle. All behavioral testing was conducted during the light period. Both the WT and CCL5^−/−^ mice were randomly assigned to the respective groups and were age-matched (4–8 month old). The housing of mice and all the animal studies were performed in accordance with protocols approved by the Institutional Animal Care and Use Committees of the Taipei Medical University (Protocol numbers: LAC-2015-0397; LAC-2019-0020). Mice were anesthetized using intraperitoneal injection of Zoletil 50 + Xylazine (Rompum) (Zoletil 50, Virbac, 66F4, 0.1 ml/ Xylazine, BAYER, 0.05 ml of 2% stock) for intracerebral (i.c.) surgery. AAV-CCL5 and AAV-mCherry were injected into the DG region of hippocampus (Bregma: 1.5 mm lateral, 2.6 mm posterior; 3 mm ventral). For both WT and KO control mice, the viral titers of AAV mCherry were (1.4 × 10^11^ VGC/ml), WT AAV CCL5 group the viral titers were AAV mCherry (1.86 × 10^10^ VGC/mL) and AAV CCL5 (1.3 × 10^12^ VGC/mL); KO AAV CCL5 group the viral titers were AAV mCherry (5.58 × 10^10^ VGC/mL), and AAV CCL5 (4 × 10^12^ VGC/mL). Injections were 0.5 microliters/min and the needle was left in position for 4 min after injection. MRI scans were conducted 3 months after surgery. At the end of analysis, the mice were sacrificed by CO_2_ necrosis and the tissues were collected for protein analysis, immunohistochemistry, electrophysiological tests, and Seahorse analysis of mitochondrial activity.

### Behavioral tests

All behavioral measurements were performed at monthly intervals. These consisted of the novel object recognition test (NOR) and the Barnes maze. All testing was analyzed via EthoVision® XT (version 12, Noldus, Boston, MA, USA) software.

(a) Novel object recognition tests (NOR): The novel object recognition test was performed before surgery, and monthly after surgery in WT and KO mice. Mice were allowed to habituate to the cage (57 cm × 57 cm box) for 2 days for a time period of 10 min each one at a time, in a soundproof room. On the third day, mice were reintroduced into the box for 10 min with two identical objects, placed at an equal distance in the box. Twenty-four hours later, one of the objects was replaced with one of the same sizes, but different in shape and color. The tracks of mouse movements were recorded and analyzed by EthoVision® XT. The preference for a new object as preference index (Time spent novel object −Time spent familiar object)/total exploration time of old plus new exploration time x 100% was used to define the mnemonic ability of mice. The combination of objects was not allowed to be repeated in a different month and the test chamber was cleaned between trials with 70% ethanol to eliminate olfactory cues.

(b) Barnes maze (BM)*:*The Barnes maze testing was performed 2–3 days after the object recognition test as previously described [58]. The maze consists of a white circular platform (92 cm of diameter) with 20 equally spaced holes at 3, 6, 9, and 12o’clock positions leading to an escape box. Mice were familiarized in a dark cage for 30 min before the experiment. In day 1, mice were habituated to the escape box for 5 min and habituated to the maze for 6 min. During the next 4 days, mice were placed on the center of maze, which they explored for 6 min. The target location was changed every month as presented in Results. Time to target, moving distance, numbers of visited error holes––“mistargets” and time spent in the target zone were measured with the software EthoVision® XT. The position of the escape box was changed each month.

### Electrophysiology study

The brain slice preparation and details of the electrophysiology study are contained in Supplementary Information.

### Golgi staining and Sholl analysis

The Golgi staining technique was used to study the morphology of the neuronal spine structures. Mice were sacrificed and the whole brains were collected and treated with the impregnation solution provided in the FD Rapid GolgiStain™ Kit (FD Neurotechnologies, Inc., Columbia, USA, Cat. #: PK401). Brains were subsequently sectioned into 150 μm slices with a Microtome (Leica, VT1000S). Slices were developed using FD Rapid GolgiStain solution and fixed on glass slides coated with gelatin (Merck) for photography. Z-stack images of prefrontal cortical neurons (40x magnification) were taken and reconstructed using Neurolucida (version 11, MBF Bioscience, Williston, VT) software. The reconstructed 2-D images were exported to Neurolucida Explorer to conduct the Sholl analysis of individual neurons with concentric circles of increasing radii (1 μm increments) centered on the cell body.

### Hippocampal tissue isolation and protein western blot analyses

Hippocampal tissues were isolated and lysed by RIPA buffer (Millipore, # 20-188, Temecula, CA, USA.) with protease-phosphatase inhibitor (Thermo Scientific, #78446, IL, USA). The synaptosome fractions were isolated by using the Syn-PER™ Synaptic Protein Extraction Reagent (Thermo Scientific, #87793). The relative protein expression of various synaptic-related and mitochondrial proteins was analyzed by Western blotting. The protein blot intensities were quantified by Image J software.

### AAV-CCL5 and AAV-mCherry packaging and preparation

The virus package and preparation are provided in Supplementary Information.

### Immunohistochemistry staining and electron microscopic analysis

Animals were transcardially perfused with 4% paraformaldehyde in phosphate buffer; brains were harvested, sectioned into 25 μm by cryostat and processed for immunohistochemistry. Brain sections were blocked with 5% normal goat serum and 3% bovine serum albumin in 0.1 M Phosphate Buffer (PB) followed by primary antibody incubation overnight and by corresponding secondary antibodies. (Antibody lists are in Supplementary Table.) Fluorescence images were captured using Tissue Gnostics Axio Observer Z1 microscope (TissueGnostics GmbH, Vienna, Austria) in Fig. 2 and Supplementary Fig. 5. Sections from various groups were reacted in the same well and controls consisted of omission of the primary antibody. Animals were perfused with 2% paraformaldehyde and 2.5% glutaraldehyde in 0.1 M PB for transmission electron microscopy; brain tissues including sensory cortex, DG, and CA1 regions were cut out and postfixed with OsO4. After washing and serial dehydration, tissues were treated with PO (propylenoxide) and Epon following by embedding into resin and sectioning into 0.38 μm. Samples were examined and photographed using TEM HT7700 (HITACHI, Tokyo, Japan).

### MRI acquisition and DTI analysis

MRI acquisition and DTI image analysis and quantification are contained in Supplementary Information.

### LC-ToF MS and metabolomic analysis

The LC-ToF MS, Metabolomic sample preparation, data analysis and quantification are contained in Supplementary Information.

### Seahorse mitochondrial activity analysis

Seahorse XF24 Extracellular Flux Assay kit (Agilent Technologies, California, USA, Lot: Q16518) was rehydrated with XF Calibrant (Lot:14418001) overnight at 37 °C. Mice were anesthetized with 3% isoflurane and decapitated rapidly. Hippocampal CA1 and dentate gyrus (DG) were isolated and cut into 1 mm × 1 mm sizes. Tissues were put onto an Islet capture microplate (lot: 21318) with aCSF (125 mM NaCl, 1.25 mM NaH_2_PO_3_, 2.5 mM KCl, 10 mM MgCl_2_, 2 mM glutamine). The basal oxygen consumption rate (OCR) and extracellular acidification rate (ECAR) in different brain tissues were detected with the Seahorse XF24 analyzer (Agilent Technologies, California, USA). The protocol was 6 min mix, 3 min wait, and 6 min measure sequences repeated six times. The middle three detection periods were averaged and normalized to the wet weight of tissue.

### NADPH oxidase activity assay

NADPH oxidase activity was measured in 4-month-old mice. Briefly, tissues were placed in a final volume of 200 µl containing Krebs buffer (pH 7.4, 118.4 mM NaCl, 25 mM NaHCO_3_, 11.7 mM glucose, 4.75 mM KCl, 1.2 mM MgSO_4_, 2.5 mM CaCl_2_.2H_2_O, 1.2 mM KH_2_PO_4_). In total, 50 µM lucigenin (Sigma-Aldrich, 2315971) was used to measure hippocampus levels detected for 50 sec as baseline. NADPH oxidase activity was assayed by adding 50 µM NADPH thereafter. Counts per second (CPS) were measured by Triathler Multilabel Test (425-004, HIDEX, Turku, Finland) and normalized with wet weight of tissue (µg).

### Quantitative PCR of gene expressions in mouse brain

Primer lists and related information are shown in the Supplementary Information.

### Primary neuron culture studies, ATP assay, immunostaining, and quantification

Hippocampal neurons were cultured from CCL5^-/-^ embryos at day 16.5 (E16.5–E17.5) [59]. In total, 4 × 10^5^ cells were seeded into 6-well plates for CCL5/RANTES stimulation studies; in parallel, 8 × 10^5^ cells were seeded in 6-well plates to observe the effects of AICAR and CCL5 in primary neurons. The cells were treated with CCL5 (10, 50, 100, and 500 pg/mL, R&D system, 478-MR-025) with an interval of 24 h for 3 days or AICAR (0.25 mM, Cell signaling, #9944) and after 30 min, treated with the different dosages of CCL5 every 24 h, for 3 days as above. On DIV 8, all the cells were collected for protein analysis, immunocytochemistry, and determination of ATP concentrations. The primary hippocampal neuron total cell lysates were collected using boiling distilled water for the quantification of ATP concentration as described in the ATP Determination Kit (Invitrogen detection technologies, Paisley, UK, Cat. #A22066) and the volume was further normalized with the protein level. For immunostaining, cultured neurons were fixed with 4% paraformaldehyde in 0.1 M PB and stained with the indicated antibody following similar protocols as used in tissues. Fluorescence images were captured using a Leica TCS SP5 confocal microscope (Leica Microsystems, Mannheim, Germany) in Fig. 4. The quantification of synaptophysin was analyzed by MATLAB (version 2019a, MathWorks, Inc, Natick, MA, USA) with SynD (version 2017) [60]. The measurement of single neurites and the co-localization of PSD 95 and Synaptophysin was done manually using the software Leica LAS AF Lite Version 2.6.0 (Leica microsystems GmbH, Wetzlar, Germany). The numbers of overlapping signals of PSD 95 and Synaptophysin were normalized by the length of the neurites. Neurons were given different treatments after 4 hr seeding and attachment, including CCL5, AICAR, MetCCL5, or PI3K inhibitor––Wortmannin (HY-10197. MedChemExpress, NJ, USA) and BLY-719 (HY-15244. MedChemExpress, NJ, USA) as well as both Wortmannin and BLY-719 with CCL5. After 24 h or 48 h, cells were fixed with 4% FPA and stained with Tau and MAP2 for axon and dendrite differentiation analysis. Neurons were also harvested for protein analysis and stained with pAkt and Tau Abs for pAkt localization analysis after 48 h.

### Quantification and statistical analysis

Statistical analysis was performed using GraphPad Prizm 8.0 (GraphPad Software, San Diego, CA, USA). An unpaired *t*-test was used to detect differences between two groups, paired *t*-test was used to compare the before and after treatment, One-way ANOVA was used to detect differences within the same group with a confidence interval of 95% and two-way ANOVA was used to detect multiple factors. The Bonferroni correction was used for serial measurements. A *p* value of <0.05 was considered significant. All results are presented as mean ± SEM.

## Supplementary information

Supplementary Information
