# Supplementary material for: CCL5 promotion of bioenergy metabolism is crucial for hippocampal synapse complex and memory formation
Source: Mol Psychiatry. 2021 Apr 30;26(11):6451–68. doi: 10.1038/s41380-021-01103-3 (PMC8760051; doi:10.1038/s41380-021-01103-3)
Supplement: Supplementary file 1 — Supplementary Information [file 41380_2021_1103_MOESM1_ESM.docx]

**Supplementary Information:**

**Includes Supplementary Materials and Methods, 13 Supplementary Figure legends, 13 Supplementary Figures**

**Supplementary Materials and Methods:**

**Materials:**

| **Antibodies** | **Source** | **Company** | **Cat. Number** | **Dilution** |
| --- | --- | --- | --- | --- |
| **Primary Antibodies** |  |  |  |  |
| NMDAR2B (NR2B) | Rabbit | Cell signal | #4212 | WB: 1:1000 |
| Phosphor-NMDAR2B S1303 | Rabbit | Millipore | 07-398 | WB: 1:1000 |
| NeuN | Mouse | GeneTex | GTX30773 | IHC: 1:200  ICC: 1:200 |
| CCL5/RANTES | Goat | R&D system | AF478 | IHC: 1:100 |
| PSD95 | Mouse | GeneTex | GTX634291 | WB: 1:1000 |
| GAP43 | Rabbit | Millipore | AB5220 | IHC: 1:100 |
| Synaptophysin | Rabbit | GeneTex | GTX100865 | WB: 1:8000  ICC: 1:500 |
| PGC1α | Mouse | Millipore | ST1203 | WB: 1:1000 |
| COX4 | Rabbit | GeneTex | GTX114330 | WB: 1:1000 |
| TOMM20 | Rabbit | GeneTex | GTX133756 | WB: 1:1000 |
| Tau-1 | Mouse | Millipore | MAB3420 | ICC: 1:400 |
| PI3 Kinase P110α | Rabbit | Cell Signaling | #4249 | WB: 1:1000 |
| Phosophor-p85 | Rabbit | Cell Signaling | #4228 | WB: 1:1000 |
| P85 total | Rabbit | Cell Signaling | #4257 | WB: 1:1000 |
| Phosophor-GSK3β | Rabbit | Cell Signaling | #5558 | WB: 1:1000 |
| Akt | Rabbit | Cell Signaling | #4691 | WB: 1:3000 |
| Phosphor-Akt T308 | Rabbit | MILLIPORE | 05-802R | WB: 1:1000 |
| Phosphor-Akt S473 | Rabbit | Cell Signaling | #4060 | WB: 1:3000  ICC: 1:200 |
| α-Tubulin | Mouse | Novus | NB100-690SS | WB: 1:10,000 |
| GFAP | Rabbit | GeneTex | GTX108711 | IHC: 1:1000  ICC: 1:1000 |
| Iba1 | Rabbit | GeneTex | GTX100042 | IHC: 1:1200  ICC: 1:2000 |
| CD11b | Rat | ThermoFisher | MA1-80091 | ICC: 1:500 |
| AMPKα total | Rabbit | Cell Signaling | #5831 | WB: 1:1000 |
| Phosphor-AMPKα T172 | Rabbit | Cell Signaling | #2535 | WB: 1:1000 |
| IRS-1 total | Rabbit | Cell Signaling | #2382 | WB: 1:500 |
| Phosphor-IRS-1 S302 | Rabbit | Cell Signaling | #2491 | WB: 1:500 |
| Phosphor-IRS-1 S612 | Rabbit | Cell Signaling | #2386 | WB: 1:500 |
| Phosphor-IRS-1 T612 | Rabbit | ThermoFisher | 44-816G | WB: 1:500 |
| MAP2 | Rabbit | Millipore | AB5622 | ICC: 1:1000 |
| Caspase 3 | Rabbit | GeneTex | GTX110543 | WB: 1:1000 |
| Caspase 9 | Rabbit | GeneTex | GTX112888 | WB: 1:1000 |
| Cleaved Caspase 9 | Rabbit | GeneTex | GTX132331 | WB: 1:1000 |
| **Secondary Antibodies** |  |  |  |  |
| Alexa Fluor 488 donkey anti-mouse IgG | Donkey | Invitrogen | A21202 | IHC, ICC: 1:400 |
| Alexa Fluor 488 donkey anti-rabbit IgG | Donkey | Invitrogen | A21206 | IHC, ICC: 1:400 |
| Alexa Fluor 568 donkey anti-rabbit IgG | Donkey | Invitrogen | A10042 | IHC, ICC: 1:400 |
| Alexa Fluor 488 donkey anti-goat IgG | Donkey | Invitrogen | A11055 | IHC, ICC: 1:400 |
| Cruz Fluor 647 donkey anti-mouse IgG | Donkey | Santa Cruz | sc-362288 | IHC: 1:400 |
| Goat anti-rat IgG-R | Goat | Santa Cruz | sc-2093 | ICC: 1:400 |
| anti-Mouse IgG | Goat | Jackson lab | 115-035-003 | WB: 1:10,000 |
| anti-Rabbit IgG | Goat | Jackson lab | 111-035-003 | WB: 1:10,000 |
| DAPI |  | Sigma | D9542 | IHC, ICC: 1:10,000 |

***Electrophysiology studies***

Hippocampal slices from the mouse brains were prepared as described previously (Chen et al, 2013). After decapitation, the brain was removed and immersed in oxygenated (95% O_2_ / 5% CO_2_) cold cutting solution (Sucrose 194 mM, NaCl 30 mM, KCl 4.5 mM, MgCl_2_ 1 mM, NaH_2_PO_4_ 1.2 mM, Glucose 10 mM, and NaHCO_3_ 26 mM). The brain tissues were cut into coronal slices (300 μm) within the chamber filled with cold cutting solution using a tissue slicer (VT 100, Leica). The brain slices were then transferred to a holding chamber filled with oxygenated artificial CSF solution (NaCl 126 mM, KCl 3 mM, MgCl_2_ 1.5 mM, CaCl_2_ 2.4 mM, NaH_2_PO_4_ 1.2 mM, glucose 11 mM, NaHCO_3_ 26 mM) at 30°C for at least 30 min to reduce endogenous adenosine levels. Extracellular recordings of field excitatory postsynaptic potentials (fEPSP) were performed in the hippocampal slices using previously described techniques ^1, 2^. Brain slices were transferred into a chamber (0.5ml, 31-33°C,) filled with aCSF with a perfusion rate of 2ml/min and/or added with different dosages of CCL5 (50, 250, 500 pg/ml). Electrical stimulation of the Schaeffer collateral pathway was performed using bipolar tungsten stimulating electrodes (Frederick Haer, Bowdoin, ME, USA) placed on the CA3 region of the hippocampus, with all of the recordings being made in the CA1 region. fEPSPs were elicited using single 0.1 ms pulses (1 –15V) delivered through the stimulating electrode at a frequency of 0.033 Hz. In each case, the fEPSP response was defined by increasing the stimulus intensity until a response that was 30% of the maximum was reached, and the voltage was left at this setting for the remainder of the experiment, to avoid ceiling effects. Data were acquired and stored on a personal computer via an A/D board (National Instruments PCI 6024E, Austin, TX, USA) using a Windows-based software package (WCP, courtesy of Dr. John Dempster, University of Strathclyde, Glasgow, UK). Analyses of amplitude and fEPSP slope of the initial (1–2 ms) rising phase of each fEPSP were performed off-line using the same software. High-frequency stimulation (HFS), consisting of three 1-sec trains of 100 Hz delivered at 10-sec intervals at twice the stimulation intensity was utilized to evoke LTP. Comparisons were then made between averages of at least 15 responses obtained during the baseline control (pre-HFS) period or at a fixed time (usually 60 min) after application of HFS.

***AAV-CCL5 and AAV-mCherry packaging and preparation***

The recombinant adeno-associated virus serotype-1 vector (AAV1-CCL5 or AAV1-mCherry, with CMV-promoter) was produced by triple plasmid transfection without the use of a helper virus. HEK 293 cells were seeded at 2 × 10^6 per 15-cm culture plate in 25 ml of DMEM plus 5% FBS for 4 days before transfection. Subconfluent (70–80%) monolayer cells were co-transfected with pAAV-CCL5 (GenBank accession number: AB051897) and pAAV-mCherry (GeneBank accession number: AY678264) (9 μg), RC1 (3.5 μg), and pHelper (12.5 μ g; Agilent Technologies) by the TransIT-X2® Dynamic Delivery System (Cat. No. MIR 6000, Mirus). A double-stranded AAV1 backbone with CMV promoter used to express mCherry, was generously provided by Dr. Brandon Harvey (National Institute on Drug Abuse, NIH). The plasmids used for transfection were purified from E. coli. Approximately 48 h post transfection, cells were harvested; the supernatants were mixed with 40% PEG8000 (in 0.9% NaCl) to a final concentration of 8%, and incubated at 4 °C overnight. Cells were lysed by freeze/thaw; the supernatant mixture was centrifuged at 4,000 × g at 4 °C for 1 hour, and the obtained pellet was dissolved in 25ml suspension buffer. Virus was purified using an AVB column (connected with P1 pump; 2 ml/min; Ref: Smith RH, ET AL. Molecular Therapy. 2009; 17:1888-1896.) and was stored at − 80 °C until use.

Viral titers were determined by a quantitative real-time PCR assay (qPCR) performed on an ABI StepOnePlus system. Five microliters of the viral sample were pre-treated with two units of DNase I (Cat. No. M0303, New England BioLabs) in a final volume of 50 μ l at 37 °C for 1 hour, and DNase I was then inactivated at 75 °C for 10 min. The primers that target WPRE on the vector plasmid (pAAV-CCL5) were used to amplify the qPCR product. Each reaction mixture (20 μl) contained 2 μ l of DNase-pretreated viral sample, 10 μl of 2 × SYBR green PCR master mix (Cat. No. K0371, Thermo), and 0.5 μ M of each primer. The PCR cycling program was as follows: 95 °C for 10 min followed by 40 cycles for amplification (95 °C for 15 sec, and 60 °C for 30 sec), and a cycle for generating a melting curve (95 °C for 15 sec, 60 °C for 1 min, and 95 °C for 15 sec). A standard curve using a ten-fold serial dilution (0.01–100 pg) of the vector plasmid (pAAV-GFP) was generated in every qPCR assay. Viral titers were expressed as viral genome copies per milliliter of the virus sample (VGC/ml) as noted above.

***MRI acquisition, DTI analysis and brain size quantification***

MRI images were acquired using a 7-Tesla scanner with a 30-cm diameter bore (Bruker Biospec 70/30 USR, Bruker Corp., Ettlingen, Germany); a linear volume coil was used to transmit the radio frequency pulses. To receive the radio frequency signal, a planar surface coil (T7399V3, Bruker Corp., Billerica, MA, USA) was placed directly over the mouse head. Each mouse was anesthetized with an isoflurane-oxygen mixture (Panion& BF biotech Inc., 2.0 vol.% with an oxygen flow of 1.2-1.4 L/min) in order to minimize motion throughout the recording session; in addition, all mice were fastened to a custom animal holder. The heating pad surrounding the abdomen of the mice kept the body temperature at 36.5 °C to 37.5 °C; a life monitoring system and pressure sensor (SA Instruments, Inc., New York, NY, USA) were placed under the animal’s abdomen to monitor the respiratory rate. Magnetic field homogeneity was optimized using standard localized shimming with first-order shims on an isotropic voxel of 7 × 7 × 7 mm^3^ encompassing the imaging slices. First, rapid acquisition with refocused echoes (RARE) T2-weighted images were acquired (TR = 2,500 ms, TE = 33 ms, matrix size = 256 × 256, field of view (FOV) = 20 × 20 mm2, 15 horizontal slices, thickness = 0.4 mm, number of excitations = 4). Subsequently, the diffusion images were acquired by DTI echo-planar imaging (EPI) Spin Echo sequence (TR/ TE = 3750 / 40.28 ms, 12 diffusion sampling directions, b-value = 1570 s/mm2, in-plane resolution = 0.4 mm, and slice thickness = 0.4 mm with 15 horizontal slices).

DTI data was analyzed by using the DSI studio (http://dsi-studio.labsolver.org), and basic analysis was performed with the following steps. First, the Allen mouse brain atlas (Lein et al., 2007) and four ROIs (dentate gyrus, hippocampus, prefrontal cortex, and somatosensory cortex) were resliced in the same dimensions as T2-weighted images by MATLAB software (https://www.mathworks.com/help/matlab/ref/rand.html). Next, the DTI images were coregistered to the reference atlas and resliced ROIs obtained the averaged fractional anisotropy (FA) in each ROI. The values of FA, which ranged from 0 (isotropy) to 1 (maximum anisotropy) were derived from the standard deviation of the three eigenvalues (λ1, λ2, λ3) of the diffusion ellipses of probability density function.

***PET imaging and analysis***

For PET imaging of cerebral glucose metabolism, 2-Deoxy-2-[18F]fluoro-D-glucose (^[18F]^FDG) was the radiolabeled compound that was used to quantify glucose metabolism in the mice using a small animal PET. ^[18F]^FDG was provided by the cyclotron facility in the National PET/Cyclotron Center (NCPP) at the Taipei Veterans General Hospital. Mice were fasted for 8–12 h before micro PET/CT and MR imaging. For the fasting condition, mice were deprived of food for 8–12 h before ^[18F]^FDG injection. Mice could access drinking water at all times. Before the PET scans, mice received the ^[18F]^FDG at a dose of 0.43–0.56 mCi dissolved in 0.3 mL of physiologic saline injected via 0.5-mL insulin syringes with a 30G needle inserted into the tail vein, and then were allowed to freely move in their cages (awake) during 45-min ^[18F]^FDG uptake. Following 45-min ^[18F]^FDG uptake, mice were placed in the PET scanner under light isoflurane anesthetic (0.5% at a flow rate of 800 ml/min) for image acquisition. A 30‐min static data acquisition was performed in the 3D list mode with an energy window of 350–650 keV in the microPET. Data were sorted into 3D sinograms, which were then single‐slice Fourier rebinned into 2D sinograms. Summation images were 3D reconstructed using a Maximum Likelihood Estimation Method (MLEM) algorithm ^3^, resulting in an image volume consisting of 240 × 240 × 31 voxels, each voxel with a size of 0.25 × 0.25 × 1.175 mm^3^.

***Quantification of ^[18F]^FDG PET and MRI data***

A co-registration technique with stand-alone PET and MRI scanners ^4^ was used to mark the specific brain regions. The implemented algorithm was based on the mutual information ^5^ paradigm, providing smaller registration errors when compared to other algorithms in the small animal brain for registration of PET to MRI ^6^. An MRI template in Allen Mouse Common Coordinate Framework (CCF) space ^7^ was created from the individual co-registered anatomical MRI datasets of all animals in this study and, together with the CT scan, was used as the basis for volume of interest (VOI) definition according to the Allen Mouse Brain Reference Atlas. Predefined segmented brain regions were used to calculate the mean regional ^[18F]^FDG uptake in hippocampi as shown in Figure 3a. The mean standardized uptake value (SUV) was obtained for each VOI. The SUV was calculated according to the injected dose and the animal’s body weight.

***Whole brain volume analysis***

For brain volumetric analysis, the whole brain area was selected by MRI analysis software, MRIcro (http://www.cabiatl.com/mricro/mricro/). The whole brain was determined according to the mouse brain atlas. The final volume calculation was based on the counted voxel numbers multiplied by the voxel size of acquired images (0.078 mm × 0.078 mm × 0.5 mm). The data of each group were then averaged.

***LC-ToF MS and Metabolomic analysis***

**Sample preparation -** Brain tissues were homogenized with water (1mg/10 μL) initially, then further diluted with five-fold (*v/v*) acetonitrile. The supernatant was recovered and dried by speed vacuum after centrifuging at 13000 rpm, 15 mins at 4°C; dry pellets were reconstituted in 150 μL of 95:5 (*v/v*) H_2_O/acetonitrile mixture.

**LC conditions -** Chromatographic separation was performed using a Waters Acquity UPLC system. The reversed-phase column used was a UPLC BEH C18 guard column (1.7 μm, 5 mm) and an analytical column (1.7 μm, 2.1 × 100 mm) operated at 45 °C. The linear gradient separations used a mobile phase consisting of A: water containing 0.1% formic acid and B: acetonitrile containing 0.1% formic acid. The gradient profile was as follows: 0–1min, 1% B; 1-15 min, 1-100% B;15-17 min, 100% B; 17-17.1 min, 100-1% B; 17.1-20 min, 1% B. The eluent was directly introduced to the MS without a split.

**MS conditions -** Mass spectrometry was performed on a SYNAPT G2 Q-ToF (Waters MS Technologies, Manchester, UK), a quadrupole and orthogonal acceleration time-of-flight tandem mass spectrometer. Simultaneous low- and high-collision energy (CE) mass spectra were collected in continuum mode over the range m/z 100–2000 every 1.2 s during the chromatographic separation. MSE parameters in positive electrospray ionization mode were as follows: capillary, 2.5 kV; sampling cone, 30 V; extraction cone, 4.0 V; desolation gas flow, 900 L/h; source temperature, 120 °C; desolation temperature, 450 °C; trap CE, off (low CE collection), trap CE ramp 15–50 V (high CE collection); lock spray configuration used the average of three m/z measurements (1.2 s scan, m/z 50–1000, every 10 s) of protonated leucine-enkephalin (m/z 556.2771) formed from infusion of a 1 μg/mL solution; this configuration typically yields mass accuracies <2 ppm. All the acquisition and analysis of data were controlled by Waters MassLynx v4.1 software.

**Metabolomics data analysis -** The metabolomics data analysis includes mass peak detection, multivariate analysis, differentially expressed metabolites (DEMs) filtering, compound identification, pathway-related compounds discovery and pathway enrichment analysis. Progenesis QI software (Waters Corporation, Milford, USA) was applied to analyze the mass raw data generated from Waters SYNPT G2 for peak alignment, peak selection and deconvolution; different parameters were adjusted for each processing step. MetaboAnalyst was used to obtain an overview and ranking of potentially important features for univariate analysis, such as fold-change and statistical testing. The heatmaps and partial least squares discriminant analysis (PLS-DA) were generated for comparison and multivariate statistical analysis. The differentially expressed metabolites (DEMs) were identified by a cutoff value greater than or equal to 2-fold-change of median intensity between two groups of samples. Progenesis QI was used for compound identification against the human metabolome database (HMDB) and evaluated the compound prediction with an overall score of 40 according to mass accuracy and isotope pattern. A cutoff value that was greater than or equal to 38 of the identified score was applied. Finally, the pathway-related compounds were determined against the KEGG database and were analyzed via MetaboAnalyst for pathway enrichment analysis.

***Nissl staining***

Mice were sacrificed and perfused with a 4% PFA solution. The brain samples were harvested and sliced in a cryostat with a thickness of 30 μM. The slices were mounted on gelatin coated slides and allowed to dry overnight to attach to the slides. The slices were washed twice in 0.1M PB for 5 minutes each and then treated with 50% ethanol (5 minutes), 75% ethanol (5 minutes), 95% ethanol (20 minutes), 75% ethanol (5 minutes), 50% ethanol (5 minutes) in order to de-fat the tissue samples and prepare them for staining. Slices were immersed in 0.1% Cresyl Violet acetate (Merck KGaA, Darmstadt, Germany, Cat. No.:10510-54-0) for 15 minutes and washed with tap water for 2 minutes. The tissues were then dehydrated in ethanol and xylene in the following order: 50% ethanol (5 minutes), 75% ethanol (5 minutes), twice in 95% ethanol (3 minutes for each wash), twice in 100% ethanol (5 minutes for each wash) and twice in Xylene (5 minutes for each wash). The slices were mounted, sealed and scanned using a TissueGnostics Axio Observer Z1 microscope (TissueGnostics GmbH, Vienna, Austria) at the Taipei Medical University Core facility.

***Apoptosis analysis in mouse brain***

For the purpose of labeling apoptotic cells in the hippocampus, the ApopTag Fluorescin In Situ Apoptosis Detection Kit (Merck KGaA, Darmstadt, Germany, Cat. No.: S7110) was used on 30μM thick brain slices. This kit utilizes the terminal deoxynucleotidyl transferase (TdT) enzyme to modify genomic DNA for the detection of apoptotic positive cells by specific staining. Images were captured using a Leica 6000 microscope.

***Seahorse mitochondria activity analysis in primary neurons and astrocytes***

The Seahorse XFe24 FluxPaks kit (Agilent Technologies, California, USA,Cat. No.: 102340-100) was used to conduct cellular studies on mitochondrial activity in neurons and astrocytes from both WT and CCL5^-/-^ mouse pups. The probes were rehydrated with XF Calibrant (Cat. No.: 100840-00) overnight at 37℃. 24 hours prior to conducting primary culture, the 24-well plate was coated with poly-l-lysine. For primary hippocampal neuronal cultures, pups were sacrificed at 16.5 days old embryonic stages and for astrocyte cultures, pups 2-3 days postnatal were sacrificed. The cells were seeded on the XFe 24 plate at a density of 2 x 10^4^ cells per well for neuron and 4 x 10^4^ cells for astrocyte cultures. The culture medium was changed at DIV 4 and the cells were treated with CCL5 (0 pg/mL. 100 pg/mL and 250 pg/mL) at DIV 5, 6 and 7. Mitochondrial activity was measured at DIV 8 using the Seahorse XFe24 Extracellular Flux Analyzer (Agilent Technologies, Santa Clara, CA, USA). The protocol used was the same as that used for tissue samples. The readings were normalized with the protein concentration of the cells in individual wells.

***Quantitative PCR analysis and gene primers:***

To assess the expression of purine-related metabolic genes, CCL5 responding receptor genes and inflammation related chemokine genes prefrontal cortex and hippocampal tissues of mice were isolated and total RNA was extracted using TRIzol® Reagent (Invitrogen). The RNA extracted from these tissue samples was then reverse-transcribed into cDNA using the SuperScript III First-Strand Synthesis System kit for RT-PCR (Invitrogen). Quantitative RT-PCR was conducted using iTaq Universal SYBR Green Supermix (Bio-Rad).

Primers used in the PCR study are listed in below:

| **Gene Name** | **Forward** | **Reverse** | **Product Size** |
| --- | --- | --- | --- |
| *Adenosine*  *Deaminase* | 5′-GAAGGTACCCAAACCAAGCA-3′ | 5′-GCTCACCCTCTGCTTTTCAC-3′ | 183 bp |
| *5'-Nucleotidase* | 5′- GATGGCTGCAAGTTCTAGGC −3′ | 5′- GACAGTAGGCGAGAGGTTGG −3′ | 237 bp |
| *Hypoxanthine-guanine phosphoribosyltransferase* | 5’-CAG GCC AGA CTT TGT TGG AT-3’ | 5’-TTG CGC TCA TCT TAG GCT TT-3’ | 147 bp |
| *CCR1* | 5′-CACCGTACCTGTAGCCC-3′ | 5′-AACCGTTCACCCACAAA-3′ | 385 bp |
| *CCR3* | 5′-CAGAGGGTGAAGAAGAC-3′ | 5′-CAACAAAGGCGTAGATT-3′ | 350 bp |
| *CCR5* | 5′-AAGAGACTCTGGCTCTTGCAG-3′ | 5′-TGAATACCAGGGAGTAGAGTGG-3′ | 152 bp |
| *GPR75* | 5′-ATGGCGATGATGACTCTAGCC-3′ | 5′-CGAGCATGTGCCAAAGAAG-3′ | 125 bp |
| *IL1-β* | 5′-GCACTACAGGCTCCGAGATGAAC-3′ | 5′-TTGTCGTTGCTTGGTTCTCCTTGT-3′ | 147 bp |
| *IL-10* | 5′-GCTCTTACTGACTGGCATGAG-3′ | 5′-CGCAGCTCTAGGAGCATGTG-3′ | 105 bp |
| *TNF-α* | 5′-GGAACTGGCAGAAGAGGCACTC-3′ | 5′-GCAGGAATGAGAAGAGGCTGAGAC-3′ | 89 bp |
| *GAPDH* | 5′-TGACATCAAGAAGGTGGTGAAG −3′ | 5′-AGAGTGGGAGTTGCTGTTGAAG −3′ | 109 bp |

**Supplementary figure legends:**

**
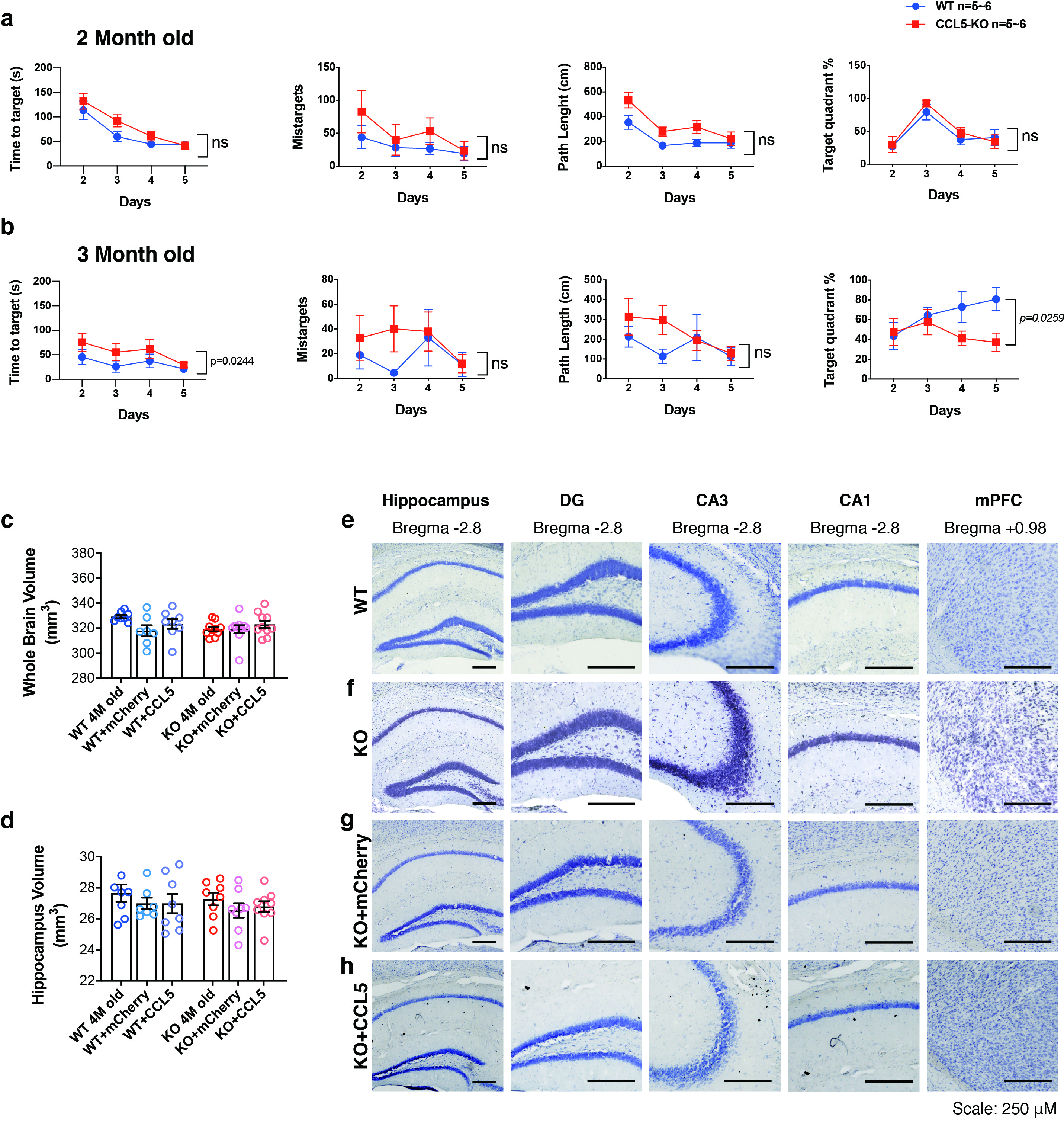
**

**Supplementary Figure 1: Hippocampus memory function and brain structure in mice.** The learning-memory performance was analyzed by Branes maze. The time to target, mistargets, path length and time in target quadrant of CCL5-KO and WT at 2-month-old (a) and 3-month-old (b) mice were analyzed (n=5~6) (Data were analyzed by two-way ANOVA). (c-d) The size of whole brain (c) and hippocampus (d) of both WT and CCL5-KO animals were analyzed from MRI images. The brain size was measured in mice 4-months old before virus injection and also 7-months old, 3-months after AAV injection (n=6~8). No differences between groups. (e-h) Nissl staining shows the distribution of neurons in different brain regions including hippocampus – DG, CA3 and CA1 and prefrontal cortex in WT, CCL5-KO, and KO with AAV-mCherry, or KO with AAV-CCL5 groups.

**
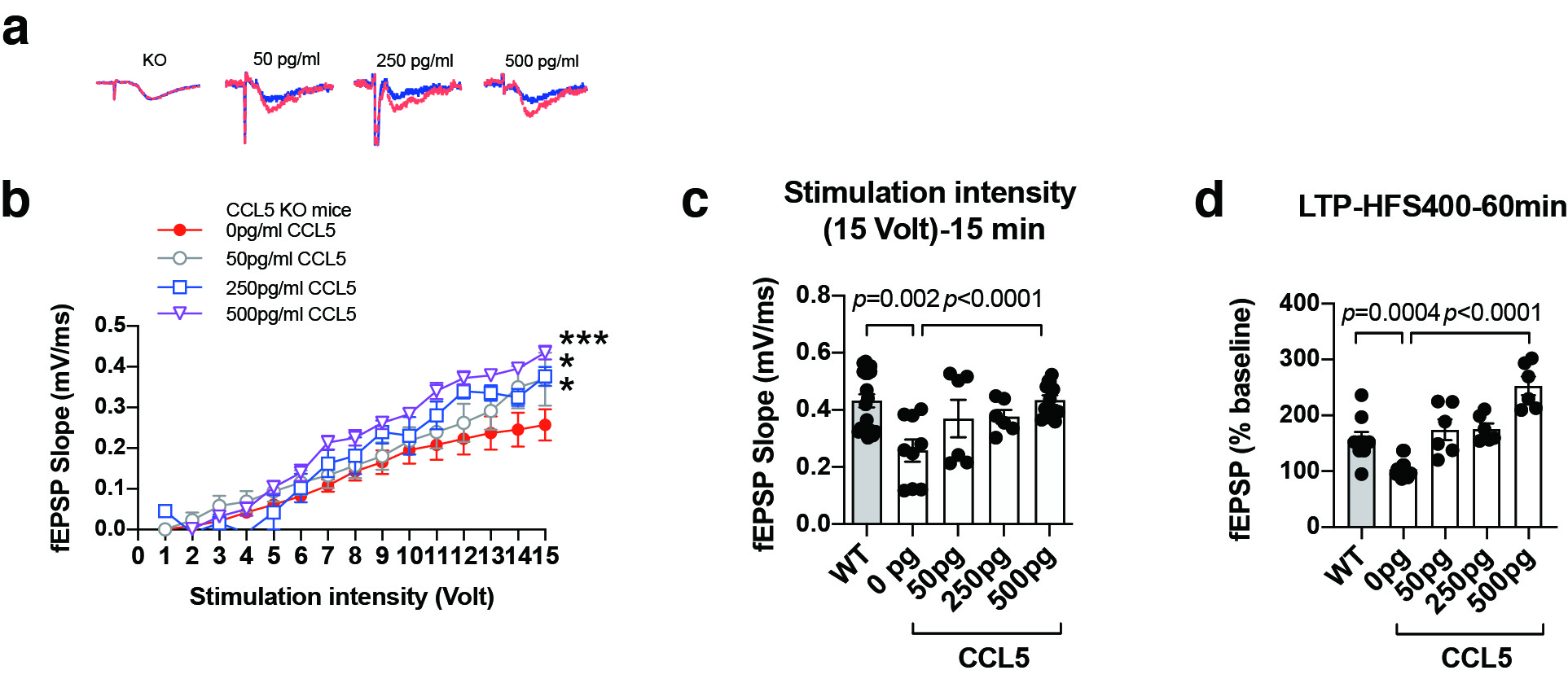
**

**Supplementary Figure 2: LTP modulated by CCL5 in KO mouse hippocampal slices.** (a) Stimulation of hippocampal slices influenced by recombinant CCL5 in CCL5-KO mice. (b) The slope of electrically stimulated fEPSPs in CCL5-KO mice upon different doses of CCL5. (Two-Way ANOVA Bonferroni's Multiple Comparison Test, *, *p*<0.05; ***, *p*<0.001. n=6~12 slices of 3 mice.). (c) The fEPSP slope with 15 volt stimulation intensity in WT and KO mice treated with different doses of CCL5. (d) The fEPSP amplitude upon high frequency stimulation in WT and KO mice treated with different doses of CCL5. (unpaired *t*-test was used between WT and KO, Ordinary one-way ANOVA test for CCL5 treatment.)

**
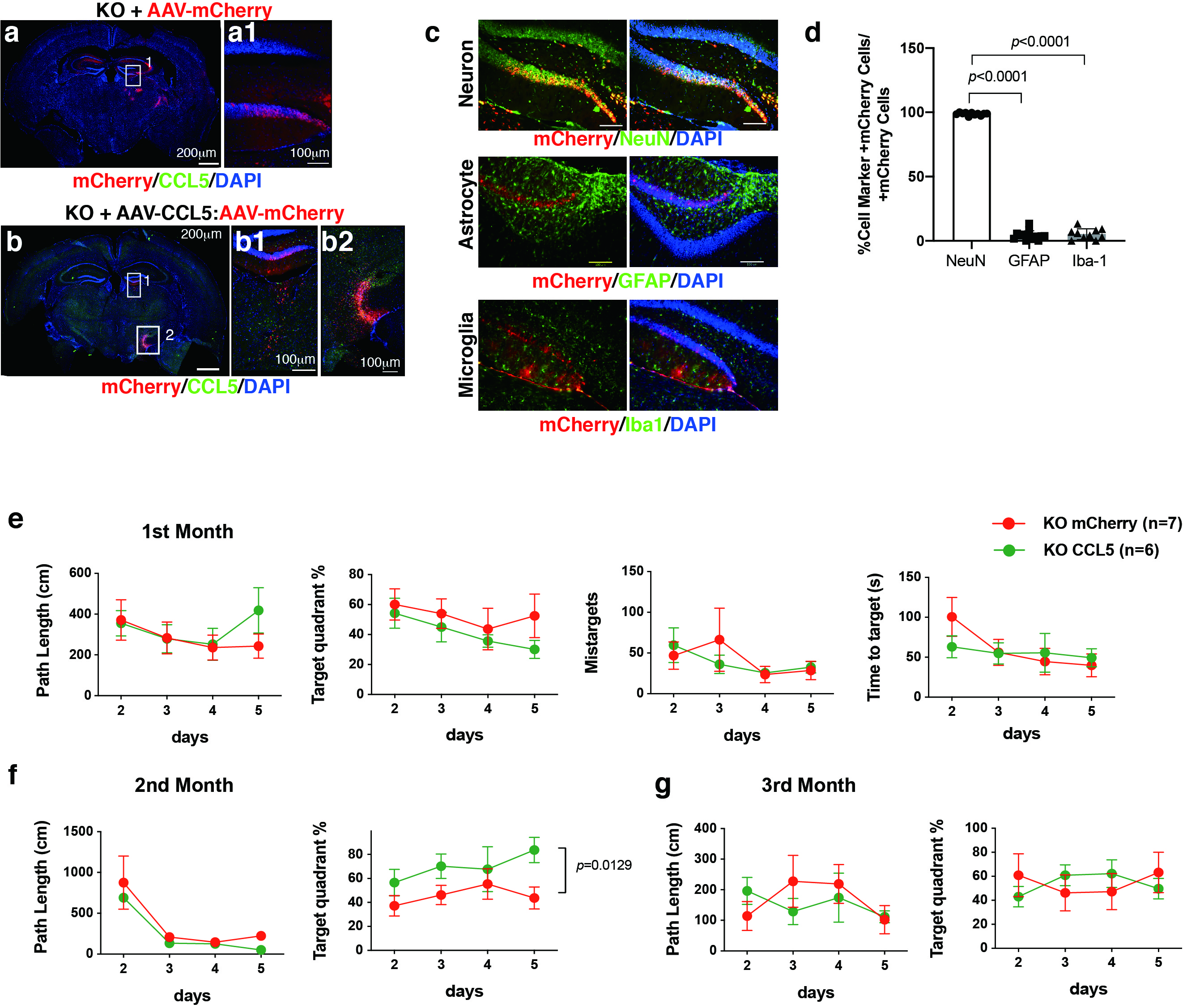
**

**Supplementary Figure 3: The distribution of proteins expressed after AAV in mouse brains and the BM performance in mice injected with AAV-mCherry or AAV-CCL5.** The distribution of AAV-mCherry (a) and AAV-CCL5 (b) in CCL5-KO mouse brains. (a1, and b1, b2 are enlarged figures from boxed regions). (c) The expression of AAV virus in neurons (NeuN co-labeled), astrocytes (GFAP co-labeled) and microglia (Iba1 co-labeled). (d) The quantification of AAV expression in different types of cells. (by unpaired *t*-test. 3 mice were used for each group and 3 brain slices from each mouse.). (e-g) The path length, time spent in target quadrant (%), number of missed targets and time to target (s) were analyzed every month for 3 months after CCL5-KO mice were injected with either AAV-mCherry or AAV-CCL5 in the BM test. The first month data is in (e), second month is in (f), and in (g) is the third month data. (e-g, Data were analyzed by two-way ANOVA.) (n= 7 for CCL5^-/-^ AAV mCherry and 6 for CCL5^-/-^ AAV CCL5 mice.)

**
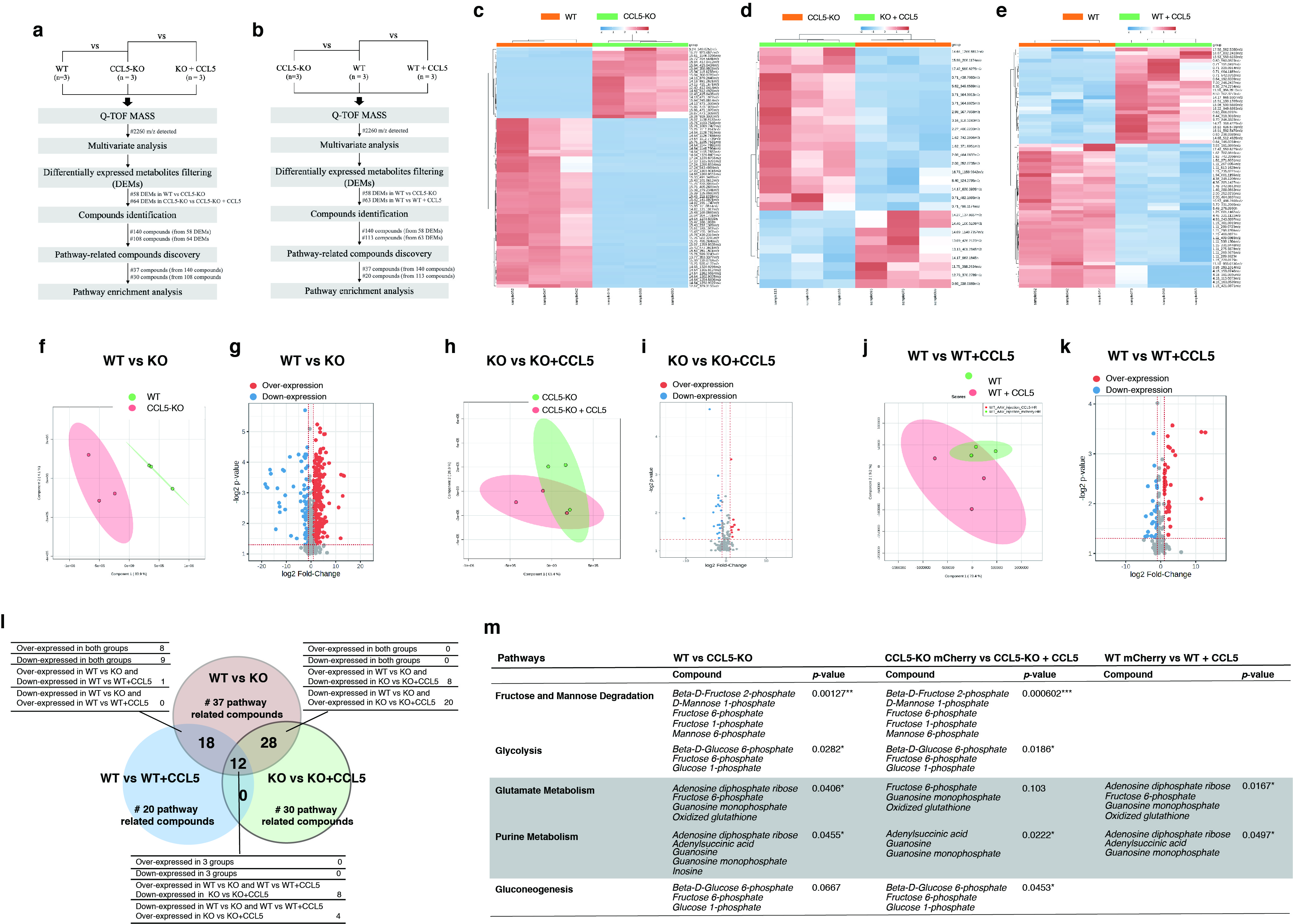
**

**Supplementary Figure 4: Metabolomics analysis in WT and CCL5-KO mouse hippocampus, and mice expressing AAV-mCherry or AAV-CCL5.** (a-b) The analysis flowchart. The heatmap of identified metabolites (m/z) between WT verses CCL5-KO (c), CCL5-KO with AAV-mCherry verses CCL5-KO with AAV-CCL5 (d) and WT with AAV-mCherry verses WT with AAV-CCL5 (e). (f, h, j) The group classification - PLSDA plot and (g, i, k) differential metabolite analysis – volcano-plot by LC Q-ToF MS from WT verses CCL5-KO, CCL5-KO mCherry verses CCL5-KO with AAV-CCL5, and WT mCherry verses WT with AAV-CCL5. (l) Pathway analysis identified 37 compounds from WT verses CCL5, 30 from CCL5-KO mCherry verses CCL5-KO with AAV-CCL5 and 20 from WT mCherry verses WT with AAV-CCL5. 28 compounds were identical in two comparisons (WT verses CCL5-KO, CCL5-KO mCherry verses CCL5-KO with AAV-CCL5), 18 compounds were identical in two comparisons (WT verses CCL5-KO, WT mCherry verses WT with AAV-CCL5), and 12 compounds were identical in three comparisons. (m) The table shows the identified compounds. Five pathways, including (1) fructose and mannose degradation, (2) glycolysis, (3) glutamate metabolism, (4) purine metabolism and (5) gluconeogenesis, have been identified between WT verses CCL5-KO, and CCL5-KO mCherry verses CCL5-KO with AAV-CCL5. (3) Glutamate and (4) purine metabolism have been identified from WT verses CCL5-KO, WT mCherry verses WT with AAV-CCL5. Gray color labeled the overlapping pathways between different comparisons. (*P*-values for each pathway were analyzed by Metaboanalyst-enrichment pathway analysis, Fisher’s exact test) (3 repeat measurements in one animal and 3 animals were used in each group.)


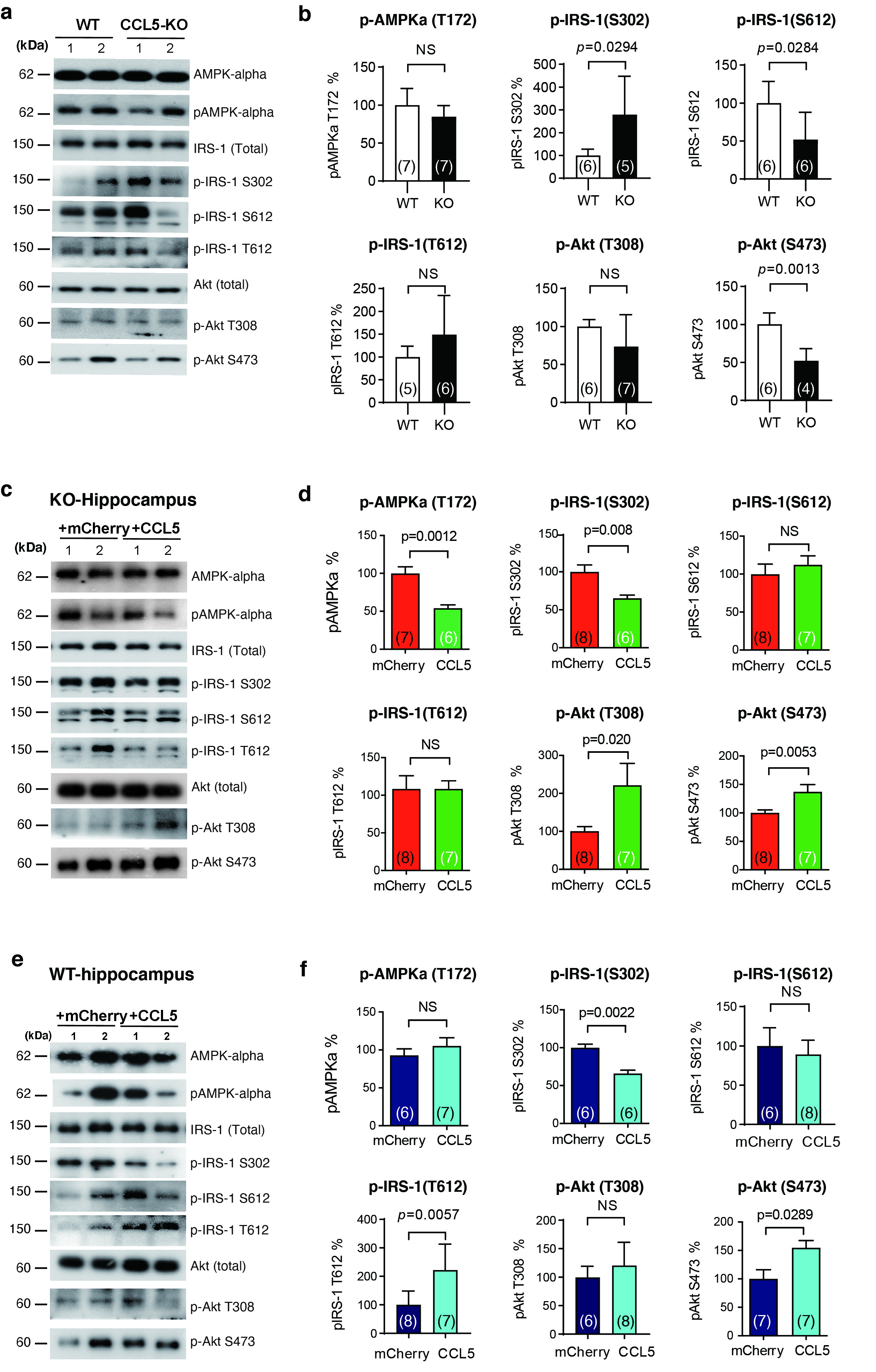


**Supplementary Figure 5: Insulin signaling pathway molecule activations in mouse hippocampus with CCL5.** The protein levels of the phosphorylated AMPKα T172, the insulin signaling pathway - phosphorylated insulin response subtract -1 (IRS-1) S302, S612, T616, and the downstream Akt phosphorylation - pAktT308 and pAktS473 were analyzed. (a) The representative protein blot images of AMPKα and insulin signaling molecule activation between WT and CCL5-KO and (b) the quantification results. (c) The protein blot images of AMPKα and insulin signaling molecule activation in CCL5-KO mice receiving AAV-mCherry or AAV-CCL5;(d) the quantification results. (e) The protein blot images of AMPKα and insulin signaling molecule activation in WT mice receiving AAV-mCherry or AAV-CCL5; (f) the quantification results. Data were analyzed by Mann-Whitney test.


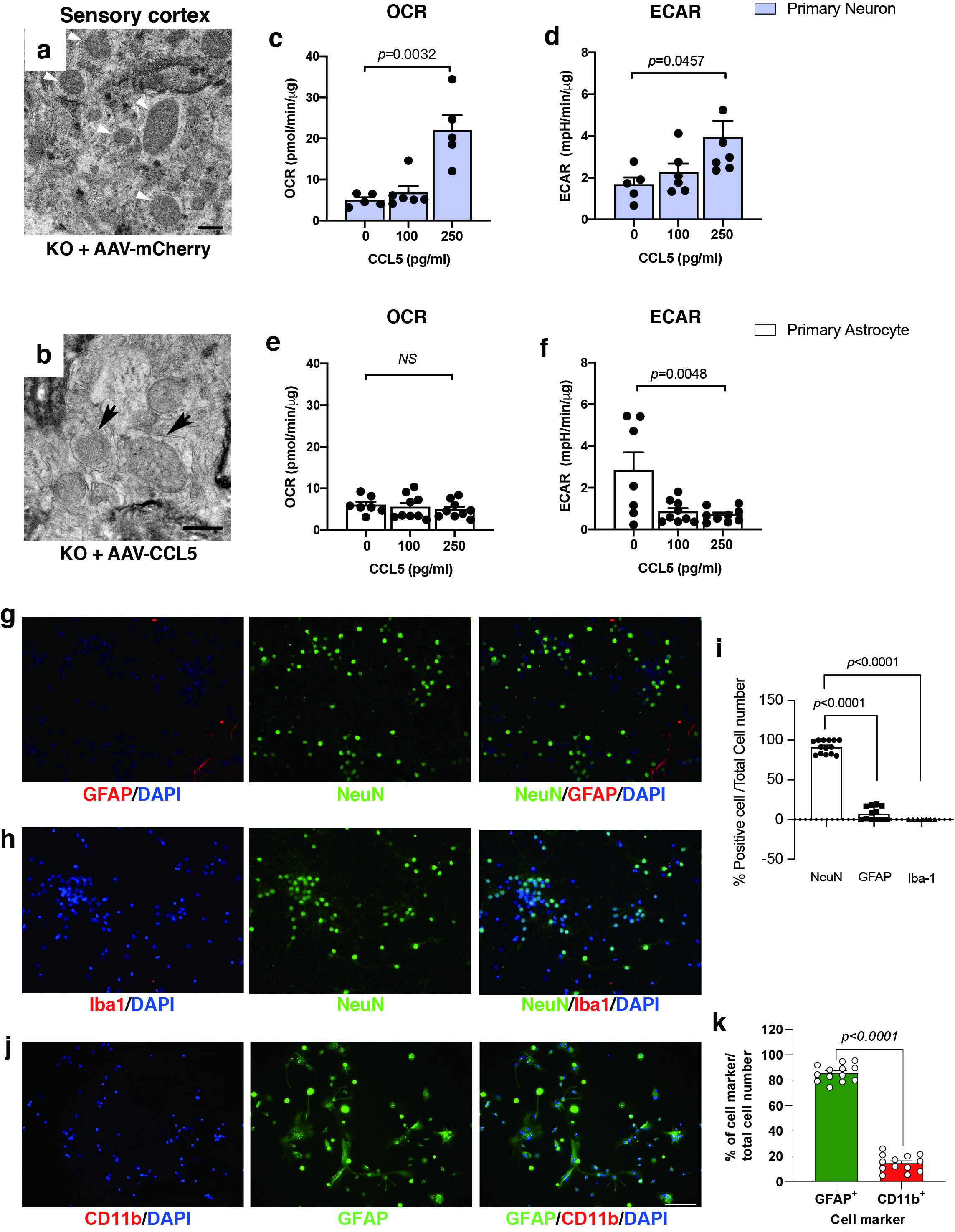


**Supplementary Figure 6: The mitochondrial structure in KO mouse somatosensory cortex and the mitochondrial activity in primary cultures of neurons and astrocytes.** The mitochondrial structure was analyzed by electron microscopy. (a, b) The mitochondria in somatosensory cortex were analyzed from the CCL5-KO mice receiving AAV-mCherry (a) (arrowheads point to the loss of outer membrane.) or AAV-CCL5 groups (b) (arrows point to the mitochondria in the autophagosome). (2~3 mice and 20 pictures were analyzed for each brain area). (c-f) The mitochondria activity - OCR and ECAR in primary neurons and astrocytes cultured from WT mice and treated with CCL5. (c-f, Data were analyzed by ordinary one-way ANOVA test. Number of repeats = 5-9 for 0 pg/mL, 100 pg/mL, 250 pg/mL CCL5 treated neuronal cultures and 0 pg/mL, 100 pg/mL, 250 pg/mL CCL5 treated astrocyte culture.) The immunostaining of astrocytes –red (GFAP) (g), microglia - red (Iba-1) (h) and neuron - green (NeuN)(g-h) in primary neuronal cultures. (i) The quantification of neuron’s astrocytes and microglia in different batches of culture. (j) The immunostaining of astrocytes –green (GFAP) and microglia - green (Iba-1) in primary astrocyte cultures. (k) The quantification of microglia and astrocyte in different batches of culture. (Data were analyzed by unpaired *t*-test in i and k.)

**
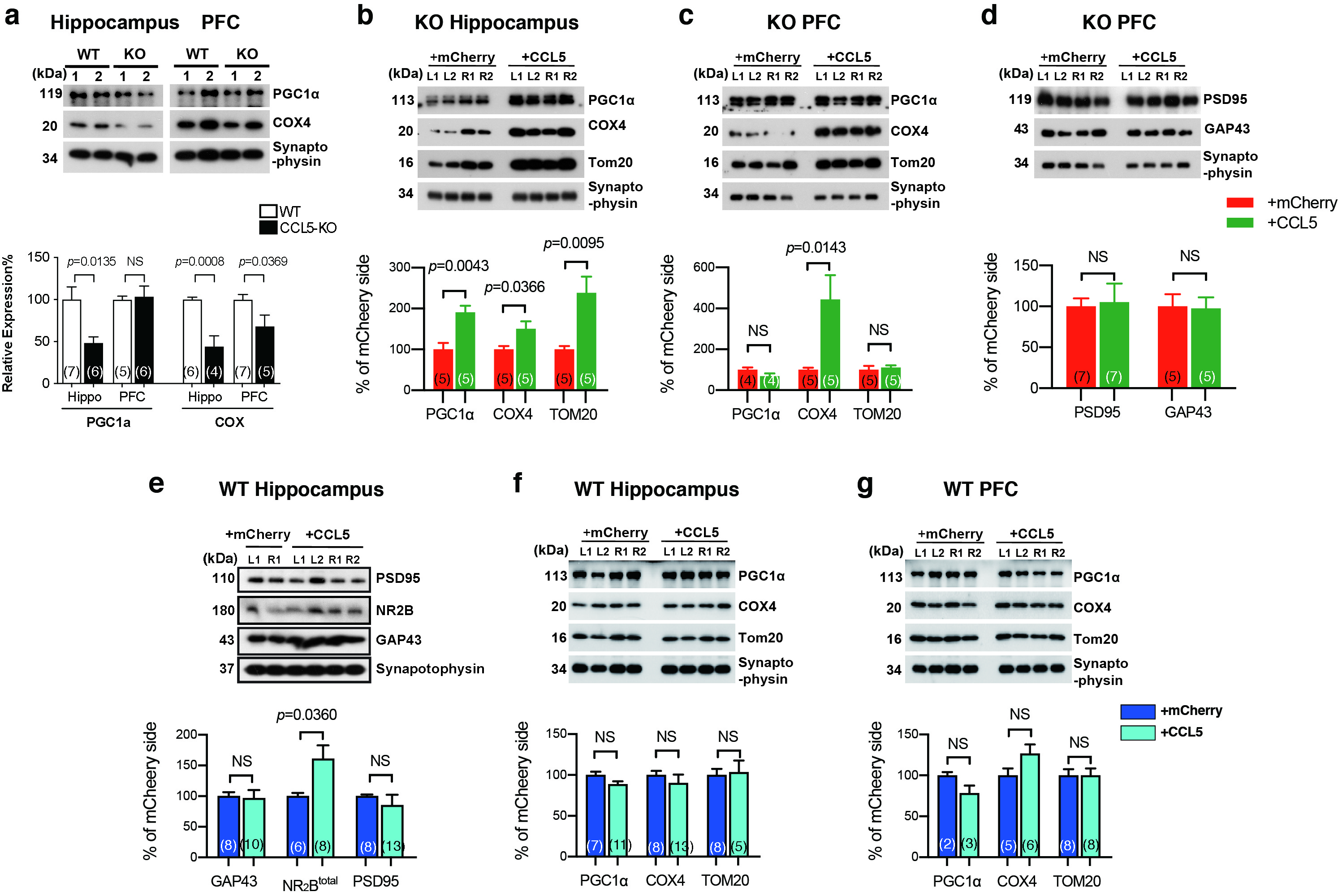
**

**Supplementary Figure 7: The protein blots and quantification of hippocampus and prefrontal cortex synaptosome fractions in WT and CCL5-KO mice with different treatments.** (a) The quantification of mitochondria proteins – PGC1-α and COX in WT and CCL5-KO mouse hippocampus and prefrontal cortex from Fig.3h. (b-c) The quantification of mitochondria protein – PGC1a, COX and Tom20 in KO mouse hippocampus (Hipp) and prefrontal cortex (PFC) after receiving AAV-mCherry or AAV-CCL5 3 months. (Protein images also see Fig.3m.) (d) The protein blot and quantification of synaptic proteins – PSD95 and GAP43 in KO prefrontal cortex (PFC) after AAV injection of mCherry or CCL5. (e) The quantification of synaptic proteins – PSD95, NR2B and GAP43 in WT hippocampus after AAV injection of mCherry or CCL5 (Protein image as in Fig.5d). (f-g) The quantification of mitochondria protein – PGC1-α, COX and Tom20 in WT mice hippocampus (Hipp) and prefrontal cortex (PFC) after receiving AAV-mCherry and AAV-CCL5 3 months. (Protein images also seen Fig.5f.) (Data were analyzed by Mann-Whitney test. n=3~5 in each group)

**
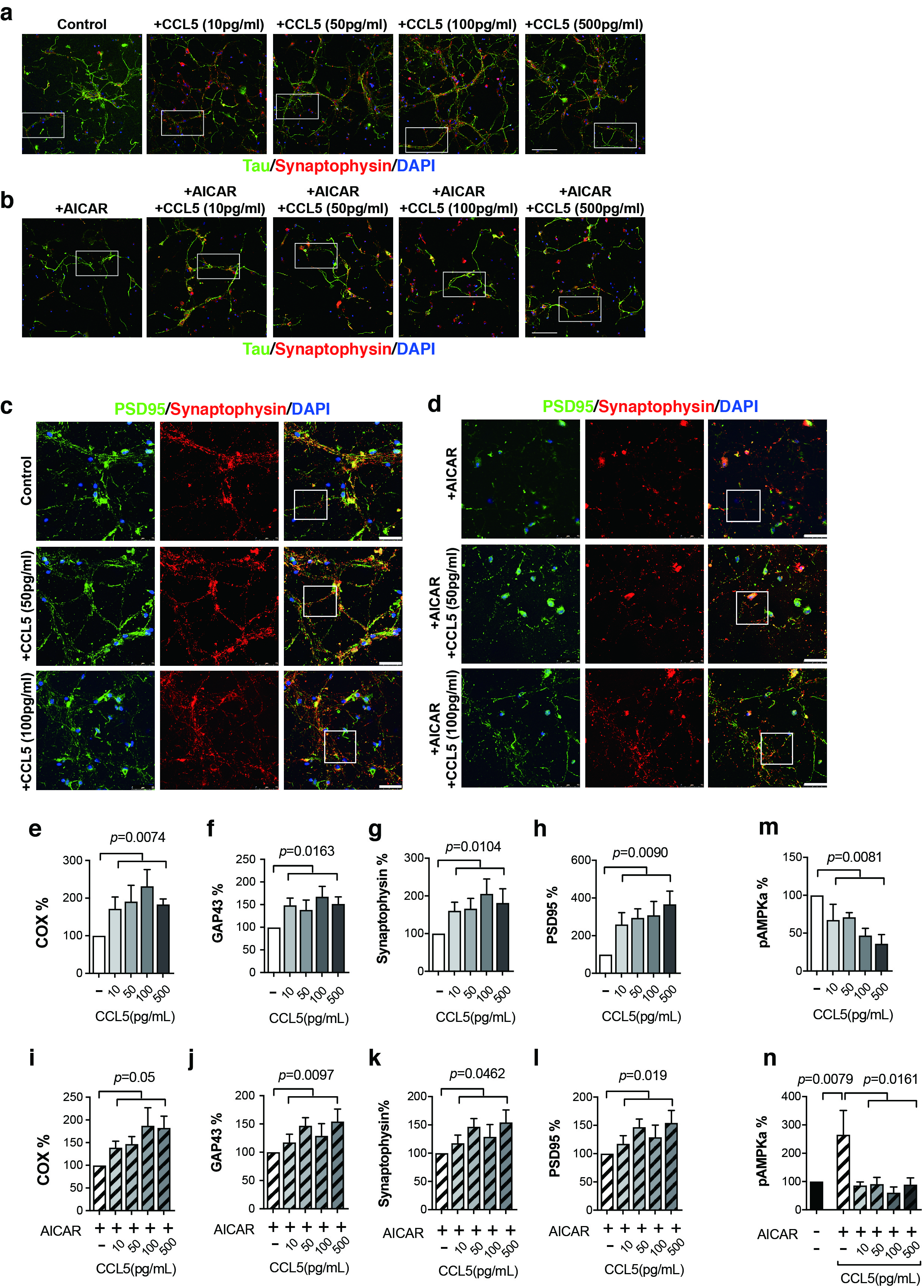
**

**Supplementary Figure 8: Synaptic complex formation and the protein levels of synaptic and mitochondrial proteins in primary hippocampal neurons treated with CCL5 and AICAR.** (a-b) The synaptic protein – synaptophysin distribution in neurons cultured with different doses of CCL5 (a) or AICAR and AICAR with CCL5 (b). Boxed regions were enlarged in Fig. 4b-c. (c-d) The colocalization of pre-synaptic protein -synaptophysin and post-synaptic protein -PDS-95 in primary neurons cultured with CCL5 (c) or AICAR and AICAR with CCL5 (d). Boxed regions were enlarged in Fig.4f-g. The quantification of mitochondria protein – COX, synapse proteins – GAP43, synaptophysin and PSD95 in primary cultured hippocampal neurons with different doses of CCL5 (0, 10, 50, 100, and 500 pg/ml) (e-h) or co-treatment with CCL5 and AICAR (0.25 mM) (i-l). The phosphorylation of AMPKα upon CCL5 (m) and AICAR (n). (n=4~6 in each group. Data were analyzed by Kruskal-Wallis test one-way ANOVA and unpaired *t*-test in (n) compared to control.)

**
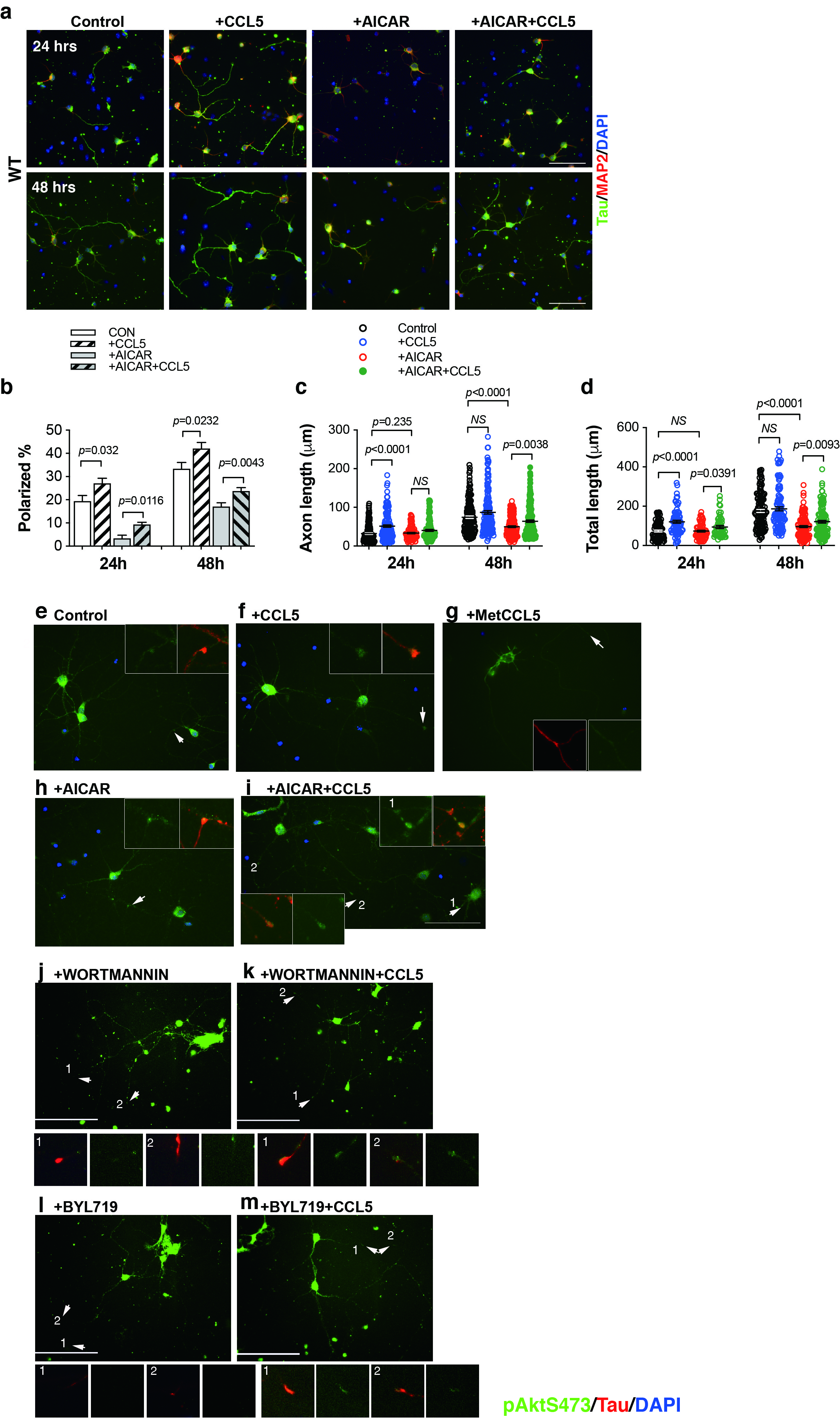
**

**Supplementary Figure 9: CCL5 and energy status in neurite polarization and elongation.** (a) WT primary neurons treated with AICAR and CCL5. Axons and dendrites were differentially labeled by Tau (green) and MAP2 (red). (b) The percentage of polarized neurons after AICAR, CCL5 and AICAR plus CCL5 treatment after 24 and 48 h were quantified. The length of axons (c) and total neurite length (d) were also measured. (Data were analyzed by Mann-Whitney test.) (e-m) Phospho-Akt S473 (green) localization was labeled by p-Akt S473 antibody and co-labeled with the axon marker -Tau (red) after (f) CCL5, (h) AICAR, (i) AICAR+CCL5, (g) antagonist ^Met^CCL5 and PI3K inhibitor – (j, l) Wortmannin and BLY179 treatment, or (k, m) Wortmannin and BLY179 with CCL5. Box regions showed the pAktS473 at neurite tip region as in Fig.4q. (Scale bar=100 μm).

**
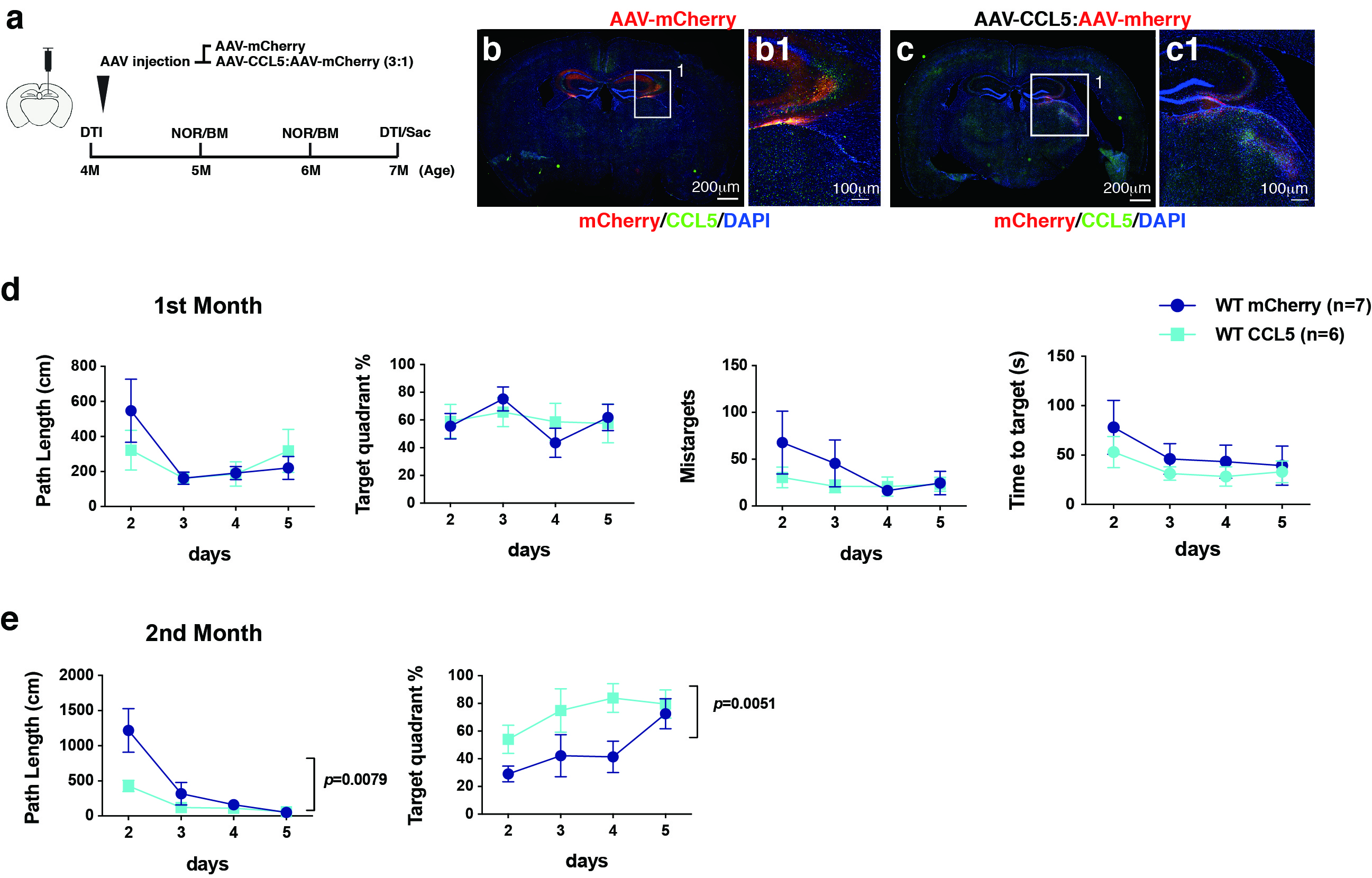
**

**Supplementary Figure 10: The AAV distribution and BM recognition performance in WT mice.** (a) The scheme of experimental design. (b-c) The AAV-mCherry (red) and both endogenous CCL5 and AAV-CCL5 (green) distribution in WT mouse brains. (b1 and c1 were enlarged from boxed regions in b and c.) DAPI labeled nucleus in blue. (d-e) The path length, time in target quadrant (%), missed target and time to find target in mice injected with AAV for one month (d) and 2 months (e). Data were analyzed by two-way ANOVA.

**
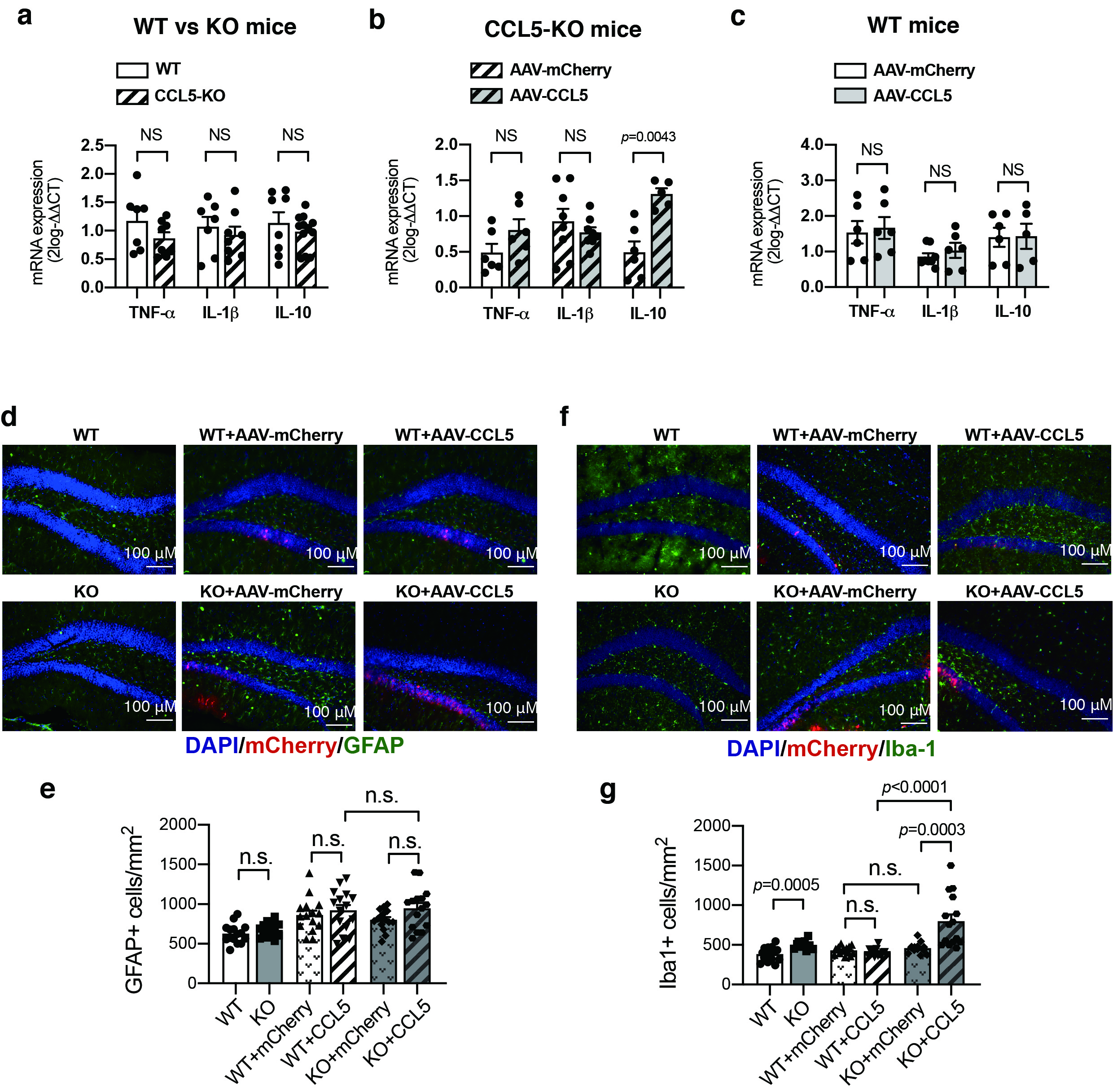
**

**Supplementary Figure 11: The expression of inflammatory genes – TNF-α, IL-1β, IL-10 and the immunostaining of astrocyte and microglial activation in mouse hippocampus.** (a-c) The quantitative PCR quantification of inflammatory genes – TNF-α, IL-1-β and IL-10 in (a) WT and CCL5-KO hippocampus (b), KO mice expressing AAV-mCherry and AAV-CCL5 and (c) WT mice expressing AAV-mCherry and AAV-CCL5. (a-c, Data were analyzed by unpaired *t*-test.) The immunostaining of GFAP - astrocytes (d) and Iba1- microglia (f) in WT and KO mice hippocampus or in WT and KO mice with AAV-mCherry and AAV-CCL5 expression. (e, g) The quantification of the number of GFAP positive astrocytes and Iba-1 positive microglia in hippocampus. (5 slices from each mouse and 3 mice from each group. Data was analyzed by unpaired *t*-test)

**
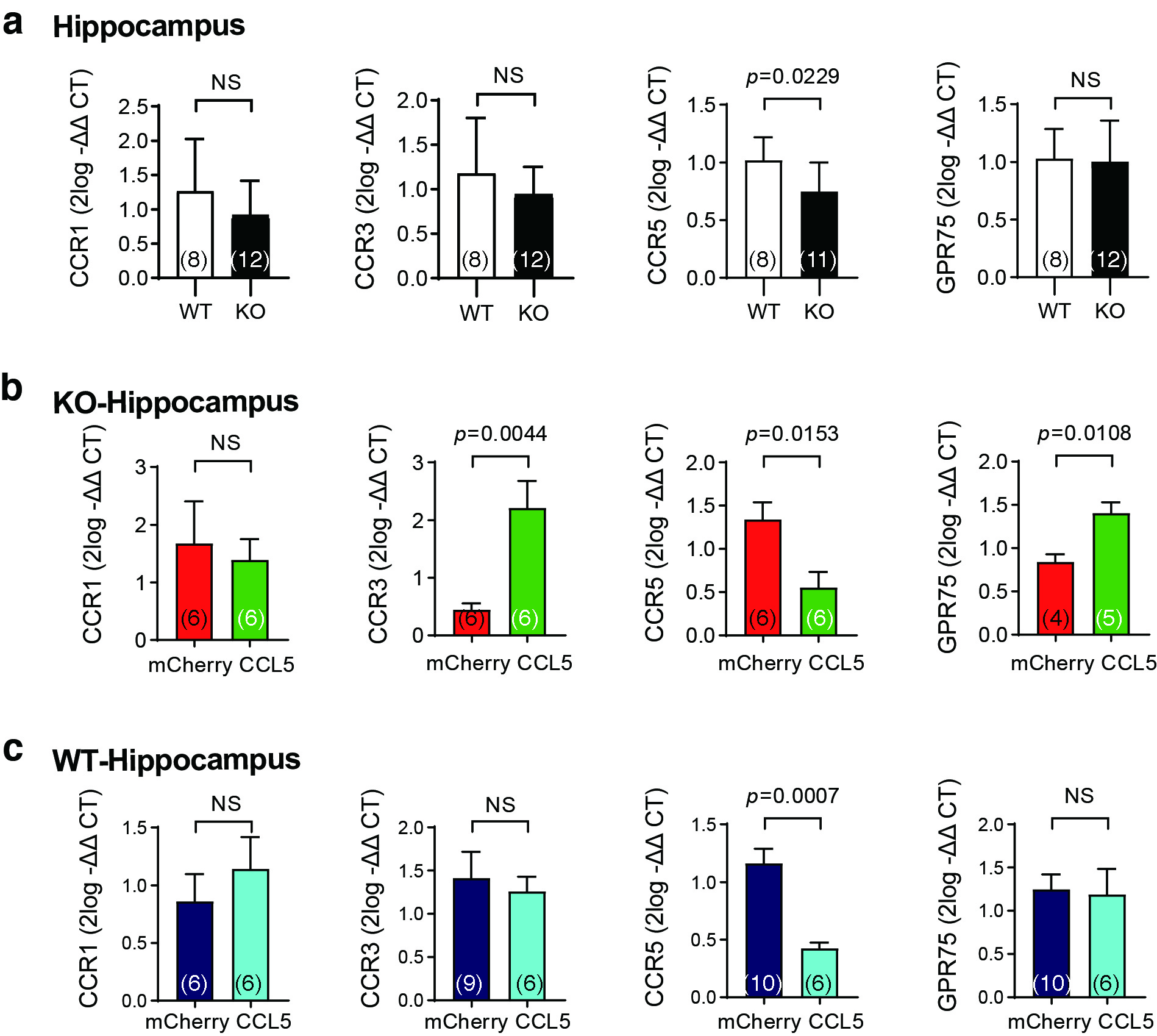
**

**Supplementary Figure 12: The expression of CCL5 related receptors – CCR1, CCR3, CCR5 and GPR75 in mouse hippocampus.** (a-c) Quantitative PCR of CCR1, CCR3, CCR5 and GPR75 in WT and CCL5-KO mouse hippocampus (a), KO mice with AAV-mCherry or AAV-CCL5 (b) and WT mice with AAV-mCherry or AAV-CCL5 (c). Data were analyzed by unpaired *t*-test.


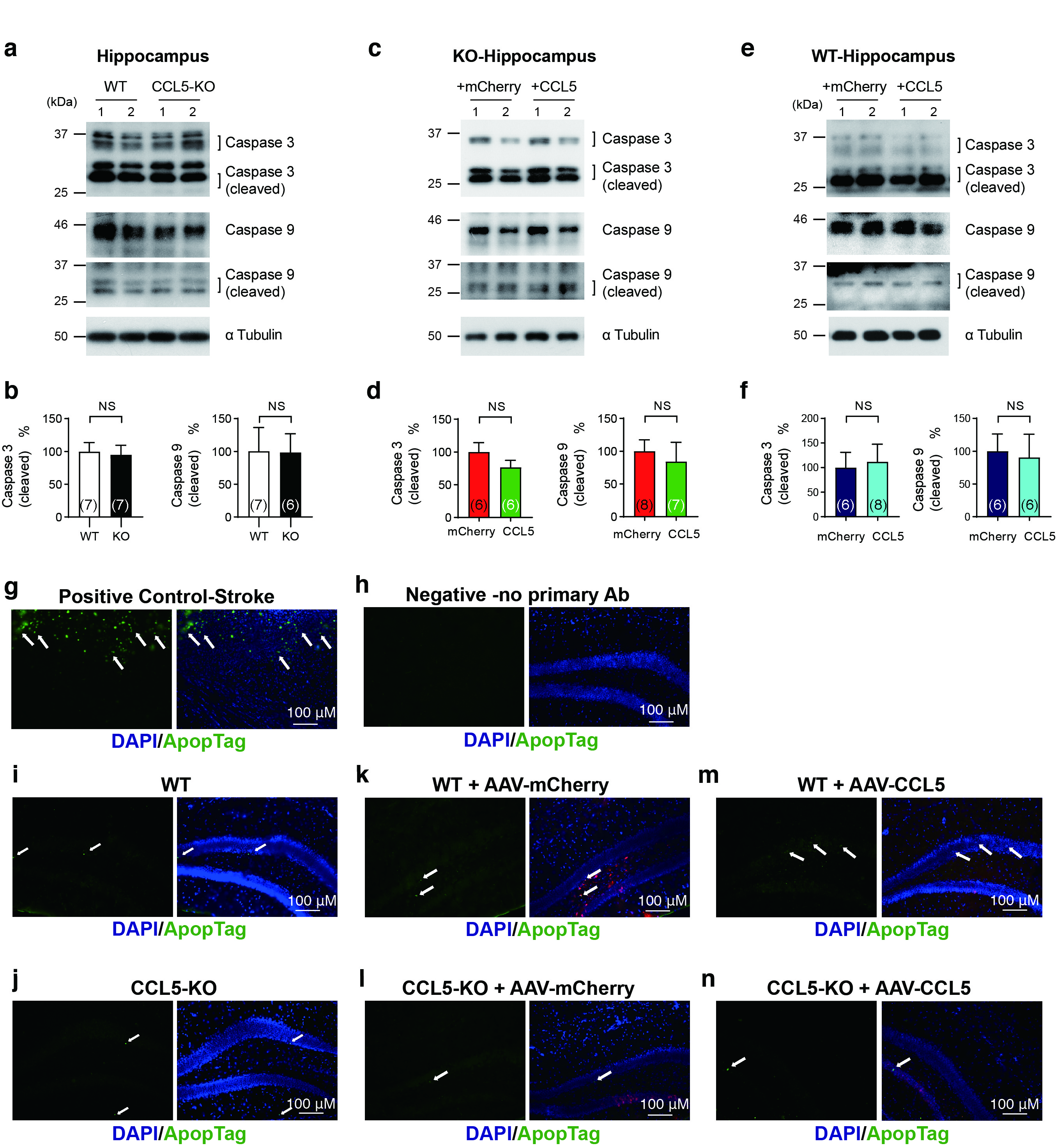


**Supplementary Figure 13: Apoptosis signaling molecule expressions and ApopTaq labeling in mouse brain.** (a-f) The expressions of cleaved Caspase 3 and cleaved Caspase 9 in mouse hippocampus were analyzed. (Data were analyzed by Mann-Whitney test.) (g-n) ApopTaq labeled apoptotic cells in mouse brain. (g) A stroke mouse brain was taken as positive control. (Green dots show apoptotic cells in the brain.) (h) Tissue labeled without primary antibody was taken as negative control. The ApopTag labeled apoptotic cells in the hippocampus region of WT (i), CCL5-KO (j), WT mouse with AAV-mCherry (k) or AAV-CCL5 (m), and CCL5-KO with AAV-mCherry (l) or AAV-CCL5 (n). Arrows point to the apoptotic cells in tissue. 1-3 mice for each group and 2 slices from each mouse were analyzed.

**Supplementary Reference:**

1. Hoffman AF, Oz M, Yang R, Lichtman AH, Lupica CR. Opposing actions of chronic Delta9-tetrahydrocannabinol and cannabinoid antagonists on hippocampal long-term potentiation. *Learn Mem* 2007; **14**(1-2)**:** 63-74.

2. Chen YH, Kuo TT, Chu MT, Ma HI, Chiang YH, Huang EY. Postnatal systemic inflammation exacerbates impairment of hippocampal synaptic plasticity in an animal seizure model. *Neuroimmunomodulation* 2013; **20**(4)**:** 223-232.

3. Myung IJ. Tutorial on maximum likelihood estimation. *J Math Psychol* 2003; **47**(1)**:** 90-100.

4. Wells WM, 3rd, Viola P, Atsumi H, Nakajima S, Kikinis R. Multi-modal volume registration by maximization of mutual information. *Med Image Anal* 1996; **1**(1)**:** 35-51.

5. Vaquero JJ, Desco M, Pascau J, Santos A, Lee I, Seidel J *et al.* PET, CT, and MR image registration of the rat brain and skull. *Ieee T Nucl Sci* 2001; **48**(4)**:** 1440-1445.

6. Xiong B, Li A, Lou Y, Chen S, Long B, Peng J *et al.* Precise Cerebral Vascular Atlas in Stereotaxic Coordinates of Whole Mouse Brain. *Front Neuroanat* 2017; **11:** 128.

7. Lein ES, Hawrylycz MJ, Ao N, Ayres M, Bensinger A, Bernard A *et al.* Genome-wide atlas of gene expression in the adult mouse brain. *Nature* 2007; **445**(7124)**:** 168-176.
